# Type I J-Domain NbMIP1 Proteins Are Required for Both *Tobacco Mosaic Virus* Infection and Plant Innate Immunity

**DOI:** 10.1371/journal.ppat.1003659

**Published:** 2013-10-03

**Authors:** Yumei Du, Jinping Zhao, Tianyuan Chen, Qi Liu, Haili Zhang, Yan Wang, Yiguo Hong, Fangming Xiao, Ling Zhang, Qianhua Shen, Yule Liu

**Affiliations:** 1 MOE Key Laboratory of Bioinformatics, School of Life Sciences, Tsinghua University, Beijing, China; 2 Research Centre for Plant RNA Signaling, School of Life and Environmental Sciences, Hangzhou Normal University, Hangzhou, China; 3 Department of Plant, Soil and Entomological Science, University of Idaho, Moscow, Idaho, United States of America; 4 Institute of Genetics and Developmental Biology, Chinese Academy of Sciences, Beijing, China; University of California, Davis Genome Center, United States of America

## Abstract

Tm-2^2^ is a coiled coil-nucleotide binding-leucine rich repeat resistance protein that confers durable extreme resistance against *Tomato mosaic virus* (ToMV) and *Tobacco mosaic virus* (TMV) by recognizing the viral movement protein (MP). Here we report that the *Nicotiana benthamiana* J-domain MIP1 proteins (NbMIP1s) associate with tobamovirus MP, Tm-2^2^ and SGT1. Silencing of *NbMIP1s* reduced TMV movement and compromised *Tm-2^2^*-mediated resistance against TMV and ToMV. Furthermore, silencing of *NbMIP1s* reduced the steady-state protein levels of ToMV MP and Tm-2^2^. Moreover, NbMIP1s are required for plant resistance induced by other *R* genes and the nonhost pathogen *Pseudomonas syringae pv. tomato* (*Pst*) DC3000. In addition, we found that SGT1 associates with Tm-2^2^ and is required for *Tm-2^2^*-mediated resistance against TMV. These results suggest that NbMIP1s function as co-chaperones during virus infection and plant immunity.

## Author Summary

Plant pathogens cause considerable yield losses in many vegetables and crops; therefore, understanding the mechanisms of disease resistance can enable crop improvements and provide substantial economic benefits. Here, we examine the signaling pathways of Tm-2^2^, a tomato resistance protein that confers resistance to *Tomato mosaic virus* (ToMV) and *Tobacco mosaic virus* (TMV) by detecting the presence of their movement proteins (MPs). We found that in *Nicotiana benthamiana*, *Tm-2^2^*-mediated resistance against TMV and ToMV requires MIP1s (NbMIP1s), a group of J-domain proteins that may act as molecular chaperones to assist protein folding and maintain protein structure. Intriguingly, MIP1s are required for both TMV resistance and TMV infection. NbMIP1s interact with and are essential for protein stability of both Tm-2^2^ and the viral MP. NbMIP1s are required for resistance mediated by other resistance proteins and resistance to the bacterial nonhost pathogen *Pseudomonas syringae pv. tomato* (*Pst*) DC3000. These new insights into the mechanisms of viral infection and disease resistance may enable future advances in crop protection.

## Introduction

Plants have evolved an effective immune system against pathogen attack. One of the most efficient plant defenses is mediated by resistance (*R*) genes [1]. The *R* gene product directly or indirectly recognizes the corresponding pathogen avirulence (Avr) protein and activates strong, specific responses that limit pathogen growth. *R*-mediated resistance is often accomplished by rapid local programmed cell death (PCD), known as the hypersensitive response (HR). However there are exceptions where no PCD is triggered [2], [3]. For instance, *Rx* from potato and *Tm-2^2^* from tomato confer extreme resistance against *Potato virus X* (PVX) or tobamoviruses *Tomato mosaic virus* (ToMV) and *Tobacco mosaic virus* (TMV), respectively, with little or no induction of visible necrotic lesions [4], [5].

Tm-2^2^ is a coiled coil-nucleotide binding site-leucine rich repeat (CC-NBS-LRR) resistance protein that confers resistance to ToMV and TMV infection by detecting the presence of their movement proteins (MPs) [6], [7]. MPs are responsible for viral cell-to-cell movement; MPs bind to viral single-stranded viral RNA to form a viral ribonucleoprotein (vRNP) complex [8], [9], and increase the plasmodesmal size-exclusion limit during viral infection [10], [11]. Two amino acid substitutions (Arg-238 and Glu-244) in ToMV MP confer the ability to overcome *Tm-2^2^*-mediated resistance [12]. *Tm-2^2^*-mediated resistance against tobamoviruses has proven remarkably durable, has been used for several decades in breeding virus-resistant tomato cultivars and has the potential to be useful in other crop cultivation [13]. Recently, the RuBisCO small subunit has been reported to be involved in both tobamovirus movement and *Tm-2^2^*-mediated extreme resistance [14]. However, the molecular pathway that leads to *Tm-2^2^*-mediated resistance remains to be elucidated.

J-domain proteins, also called DnaJ proteins, are obligate cochaperone partners of the Hsp70 chaperone [15]. J domains are conserved ∼70 amino acid motifs that are found in Hsp70 co-chaperones such as DnaJ (Hsp40). DnaJ was originally identified in *E. coli* and interacts directly with DnaK and GraE, two important components in the molecular chaperone machinery [16], [17]. J-domain proteins can bind Hsp70 to stimulate ATP hydrolysis, and stabilize the Hsp70 interaction with substrate proteins [16], [18], [19], [20], [21]. J domain proteins are involved in a variety of essential cellular processes including protein folding, assembly, translocation, degradation, stabilization and refolding [22], [23], [24], [25]. In addition to their co-chaperone activity, DnaJ proteins function as protein disulfide isomerases to catalyze protein disulfide formation, reduction, and isomerization [26].

The large and diverse group of plant J-domain proteins can be classified into four types (I, II, III, and IV) [23]. Type I J-domain proteins contain four domains including a J-domain, a Gly/Phe (G/F) domain, a (CxxCxGxG)_4_ zinc finger domain, and a less conserved C-terminal domain, whereas Types II, III and IV proteins lack one or more of these domains. Recently, it has been reported that overexpression of the type III J domain protein HSP40 causes HR-like cell death [27].

In this study, we report that *Nicotiana benthmiana* MIP1s (NbMIP1s), a group of type I J-domain proteins, associate with tobamovirus MP, Tm-2^2^ and SGT1 *in vitro* and *in vivo*, and are required for both virus infection and plant immunity by functioning as co-chaperones to maintain protein stability.

## Results

### Identification of NbMIP1s as ToMV MP-Interacting and Tm-2^2^-Interacting Partners

The tobamovirus movement protein (MP) is required for viral cell-to-cell movement and acts as an avirulence factor for *Tm-2^2^*-mediated resistance against ToMV and TMV. The LRR domain of Tm-2^2^ (Tm-2^2^-LRR) has been implicated in ToMV recognition during the virus resistance response [28]. To identify host factors that interact with ToMV MP or Tm-2^2^-LRR, we performed two yeast two-hybrid screens. ToMV MP and Tm-2^2^-LRR were used as bait to screen a tomato cDNA library [29], [30]. In these yeast two-hybrid screens, we identified RbCS as interacting with ToMV MP [14] and SGT1 [29] as interacting with Tm-2^2^. We also found that ToMV MP and Tm-2^2^-LRR interacted with one common protein, designated MIP1 (MP-Interacting Protein 1). The *MIP1* gene from our tomato cDNA library encodes a putative type I J-domain protein (The Institute for Genomic Research: TC192697, designated SlMIP1). Bioinformatics analysis identified 7 SlMIP1 homologues in the *Solanum lycopersicum* genome (http://solgenomics.net). Further, we identified 13 putative MIP1 homologs that potentially encode proteins with more than 40% amino acid identity with SlMIP1 in the *Nicotiana benthamiana* (*Nb*) genome (http://solgenomics.net). Because the predicted cDNA sequences of most *Nb* MIP1-like genes have much shorter open reading frames than those of the corresponding tomato MIP1-like genes, we believe that their cDNA sequences have not been correctly predicted. Thus, we tried RT-PCR with gene-specific primers to amplify and clone the cDNAs of all 13 putative *Nb MIP1* homologs (*NbMIP1Hs*) from total cDNA of *N. benthamiana* leaf tissues. However, we were only able to detect and clone 7 of them, suggesting that the other 6 genes are not expressed, or are rarely expressed in leaf tissues. Among these 7 NbMIP1Hs, six of them interact with ToMV MP in yeast and are called NbMIP1s whilst the other one is named NbMIP1L1 (NbMIP1-like 1) (see below). NbMIP1s can be further divided into 4 subgroups [NbMIP1.1 (a and b), NbMIP1.2, NbMIP1.3 and NbMIP1.4 (a and b)] based on their amino acid (aa) and nucleotide sequence similarity. *NbMIP1.1a* and *NbMIP1.1b* encode 420 and 418 aa proteins sharing extensive aa identity to each other (98.3%) and to SlMIP1 (95.7% and 96.4%, respectively). *NbMIP1.2* and *NbMIP1.3* encode proteins sharing 88.4% and 91.9% aa identity to NbMIP1.1a respectively. *NbMIP1.4a* and *NbMIP1.4b* encode proteins sharing extensive aa identity to each other (97%) and to NbMIP1.1a (86% for both), but NbMIP1.4a is 15 aa shorter than NbMIP1.4b, perhaps due to alternative splicing. *NbMIP1L1* encodes a 423 aa protein sharing 73.3% aa identity to NbMIP1.1a (Figure S1). However, *NbMIP1s* in different subgroups do not share any similarity at their 3′-untranslated region (UTR), although 3′-UTR sequences between two *NbMIP1s* in same subgroup share more than 90% nucleotide sequence identity. Because *N. benthamiana* is an allotetraploid, and two NbMIP1s in same subgroup share high DNA and protein sequence identity, we believe that two NbMIP1s in same subgroup (*NbMIP1.1a* and *NbMIP1.1b*; *NbMIP1.4a* and *NbMIP1.4b*) are the alleles of one gene from different ancestry. In this study, we focused on *NbMIP1.1a*.

### NbMIP1.1a Interacts Directly with Both ToMV MP and Tm-2^2^

**Figure 1:**
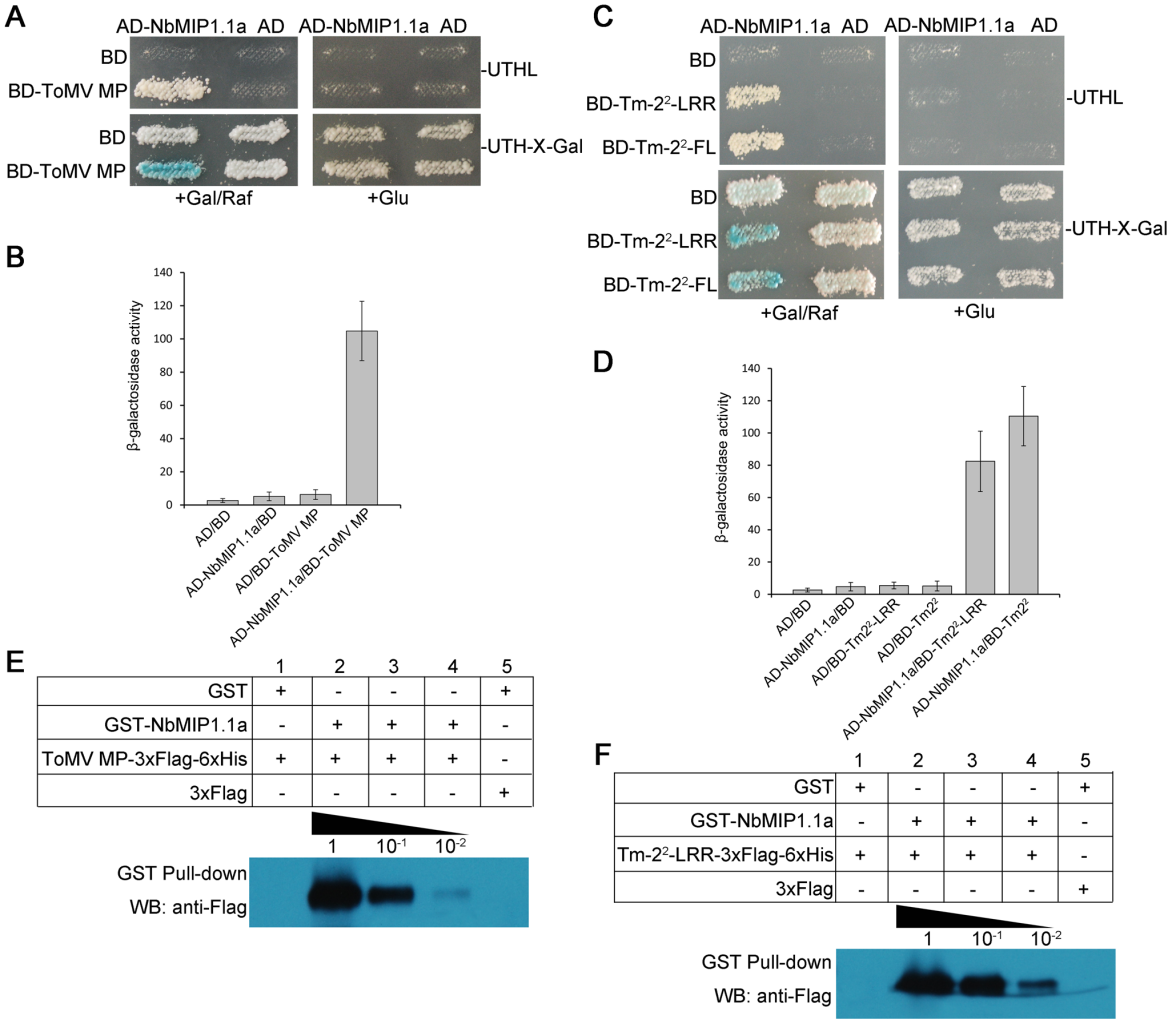
NbMIP1.1a interacts with ToMV MP and Tm-2^2^ in yeast and *in vitro*. (**A–D**) NbMIP1.1a interacts with ToMV MP, LRR domain of Tm-2^2^ (Tm-2^2^-LRR) and full length Tm-2^2^ (Tm-2^2^-FL) in yeast. (**A, C**) Yeast cells harboring BD-ToMV MP (A), BD-Tm-2^2^-LRR or BD-Tm-2^2^-FL (C) transformed with AD-NbMIP1.1a grew on Leu^-^ selection plates and turned blue on X-gal plate containing Gal/Raf but not on plates containing glucose. Yeast cells transformed with either BD or AD alone vector showed no growth on Leu^−^ selection plates and remained white on X-gal plates containing either Gal/Raf or glucose. For each experiment, yeast strains were maintained at 28°C for 5 days. (**B, D**) Quantification of β-galactosidase activities in yeast two-hybrid interactions. (**E, F**) GST pull-down assay for detection of *in vitro* interaction of NbMIP1.1a with ToMV MP (E) or Tm-2^2^ LRR domain (F). For GST pull-down, GST-NbMIP1.1a or GST immobilized on glutathione-Sepharose beads was incubated with gradient dilutions of *E. coli*-expressed recombinant ToMV MP-3×Flag-6×His protein (E) and Tm-2^2^-LRR-3×Flag-6×His protein (F) at 1, 10^−1^, 10^−2^. Beads were washed and proteins were analyzed by SDS-PAGE and western blot assays using anti-Flag antibodies.

We tested the interaction between NbMIP1.1a and ToMV MP using a yeast two-hybrid system. NbMIP1.1a was expressed as a fusion to the B42 activation domain (AD) (AD-NbMIP1.1a) and ToMV MP was expressed as a fusion to the LexA DNA binding domain (BD) (BD-ToMV MP). Expression of these fusion genes was under the control of galactose-inducible promoters. Yeast transformed with AD-NbMIP1.1a and BD-ToMV MP grew on Leu^-^ plates containing galactose, turned blue on 5-bromo-4-chloro-3-indolyl-D-galactoside (X-gal) plates containing galactose/raffinose (Gal/Raf) but not glucose and showed high levels of β-galactosidase activity (Figure 1A–1B). However, control yeast with the BD or AD alone did not grow on selection plates or turn X-gal blue on glucose or Gal/Raf or show significant levels of β-galactosidase activity (Figure 1A–1B). NbMIP1.1a therefore interacts with ToMV MP in yeast. Similarly, we found that NbMIP1.1a interacts with Tm-2^2^-LRR domain as well as the full-length Tm-2^2^ protein (Figure 1C–1D).

We then confirmed the interaction of NbMIP1.1a with ToMV MP and Tm-2^2^ *in vitro* by GST pull-down assays. For this purpose, NbMIP1.1a was bacterially expressed as a GST-tagged fusion GST-NbMIP1.1a (Figure S2A) and ToMV MP was expressed as double-tagged fusion ToMV MP-3×Flag-6×His (Figure S2B). Different dilutions of 3×Flag-6×His-tagged proteins were subjected to pull-down assay and western blot detection with anti-Flag antibodies. Western blot assays indicated that GST-NbMIP1.1a, but not GST alone, binds directly to ToMV MP-3×Flag-6×His (Figure 1E). However, we failed to express the full-length Tm-2^2^ in *E. coli*; therefore, as an alternative, we tested whether NbMIP1.1a would interact with Tm-2^2^-LRR. GST pull-down results showed that GST-NbMIP1.1a indeed bound directly to Tm-2^2^-LRR-3×Flag-6×His, but GST alone not (Figure 1F; Figure S2B). These results confirm that NbMIP1.1a directly interacts with both ToMV MP and Tm-2^2^.

### The C-Terminal Domains of NbMIP1.1a Are Responsible for the Interactions with ToMV MP and Tm-2^2^

NbMIP1s contain four domains, including an N-terminal J domain, a G/F domain, a distal zinc finger domain and a less well-conserved C-terminal domain (CTD). To examine the contributions of these domains, we focused on a typical MIP1, NbMIP1.1a. To determine the domain(s) responsible for NbMIP1.1a binding to ToMV MP or Tm-2^2^, we generated a series of NbMIP1.1a deletion mutants according to its predicted structural model (Figure S3A). The yeast two-hybrid assays demonstrated that the C-terminal region (zinc finger-CTD) of NbMIP1.1a (amino acid residues 121 to 420) interacts with ToMV MP and Tm-2^2^; by contrast, no interaction was observed between other NbMIP1.1a domains and ToMV MP or Tm-2^2^ (Figure S3B–C). These results show that the C-terminal zinc finger-CTD of NbMIP1.1a is responsible for its interaction with ToMV MP and Tm-2^2^.

### NbMIP1.1a Interacts with Both Tm-2^2^ and ToMV MP *In Vivo*

**Figure 2:**
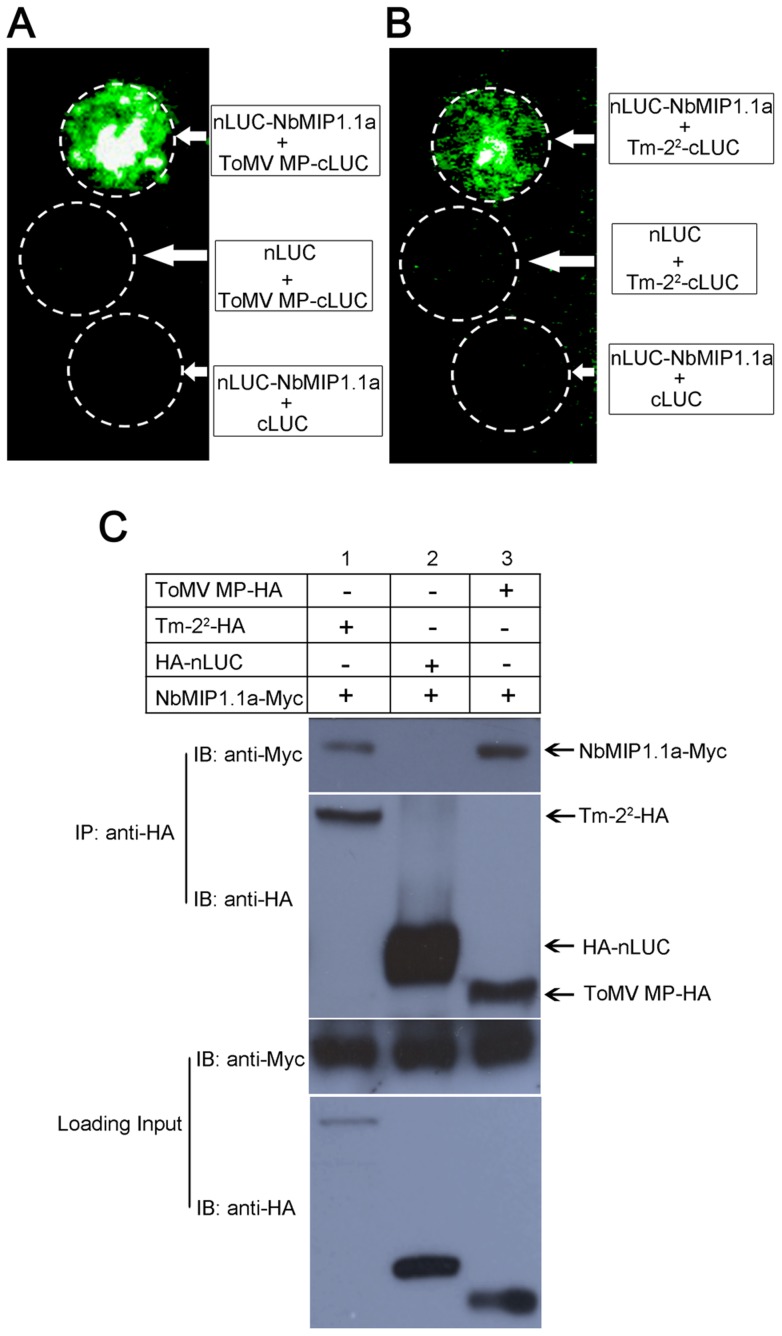
NbMIP1.1a interacts with ToMV MP and Tm-2^2^ *in vivo*. Firefly luciferase complementation imaging assays for *in vivo* interaction of NbMIP1.1a with ToMV MP (**A**) and Tm-2^2^ (**B**). Panels show luminescence images of *N. benthamiana* leaves agro-infiltrated with nLUC-NbMIP1.1a and ToMV MP-cLUC or Tm-2^2^-cLUC. The combinations of nLUC-NbMIP1.1a and cLUC, nLUC and ToMV MP-cLUC, nLUC and Tm-2^2^-cLUC were included as negative controls. (**C**) NbMIP1.1a co-immunoprecipitated (co-IP) with ToMV MP and Tm-2^2^. NbMIP1.1a-Myc was co-expressed with ToMV MP-HA or Tm-2^2^-HA in *N. benthamiana* leaves by agroinfiltration. NbMIP1.1a-Myc co-expressed with HA-nLUC was introduced as a negative control. At 48 hours post infiltration (hpi), leaf lysates were immunoprecipitated with anti-HA beads, then the immunoprecipitates were assessed by western blotting using anti-Myc (upper panel) and anti-HA antibodies (middle panel). In addition to immunoblotting for co-IP, presence of NbMIP1.1a-Myc, ToMV MP-HA, Tm-2^2^-HA and HA-nLUC in the cell lysates were also analyzed (lower panel).

To test whether NbMIP1s interact with Tm-2^2^ and ToMV MP in plant cells, we performed firefly luciferase complementation imaging (LCI) assays [31]. In this assay, the N- (nLUC) and C-terminal (cLUC) fragments of the firefly luciferase reconstitute active luciferase only when fused to two interacting proteins [31]. We generated nLUC-NbMIP1.1a and ToMV MP-cLUC fusion constructs and transiently co-expressed them in *N. benthamiana*. Positive signals were detected for the combination of nLUC-NbMIP1.1a with ToMV MP-cLUC (Figure 2A). By contrast, no signals were detected for the combination of nLUC-NbMIP1.1a with the cLUC control, or for the combination of the nLUC control with ToMV MP-cLUC (Figure 2A). Similarly, we transiently co-expressed nLUC-NbMIP1.1a and Tm-2^2^-cLUC in *N. benthamiana*. A positive signal was detected for the combination of nLUC-NbMIP1.1a with Tm-2^2^-cLUC but not for the controls (Figure 2B). In addition, LCI assays also indicated that NbMIP1.1a interacts with TMV MP in plant cells (Figure S4), consistent with the fact that MPs are much conserved among tobamoviruses (90% identity and 97% similarity between ToMV and TMV) [14].

We confirmed the *in vivo* interaction of NbMIP1.1a with ToMV MP and Tm-2^2^ by co-immunoprecipitation (Co-IP) assays. The HA-tagged ToMV MP (ToMV MP-HA) or HA-tagged Tm-2^2^ (Tm-2^2^-HA) constructs were transiently co-expressed with a Myc-tagged NbMIP1.1a (NbMIP1.1a-Myc) under the control of the CaMV 35S promoter in *N. benthamiana*. Plant leaf tissues were collected at 48 hours post infiltration (hpi) from leaves co-infiltrated with *Agrobacterium* carrying the NbMIP1.1a-Myc expression cassette together with *Agrobacterium* carrying either the control HA-nLUC, ToMV MP-HA or Tm-2^2^-HA expression cassettes. Total protein extracts were immunoprecipitated using anti-HA antibodies and the resulting precipitates were analyzed by western blot using anti-Myc antibodies. We observed that NbMIP1.1a co-immunoprecipitated with both ToMV MP and Tm-2^2^, but not with HA-nLUC (Figure 2C). These experiments, along with our LCI assays, demonstrate that NbMIP1.1a interacts with ToMV/TMV MP and Tm-2^2^ in plant cells.

### Other NbMIP1s Also Associate with ToMV MP and Tm-2^2^

We also tested the interaction of other NbMIP1s with Tm-2^2^ or ToMV MP. Yeast two-hybrid and LCI assays indicated that all other NbMIPs (NbMIP1.1b, NbMIP1.2, NbMIP1.3, NbMIP1.4a, NbMIP1.4b), but not NbMIP1L1, interact with Tm-2^2^ and ToMV MP in both yeast and plant (Figure S5).

### Tm-2^2^, NbMIP1.1a and ToMV MP May Exist in the Same Complex in Plants

**Figure 3:**
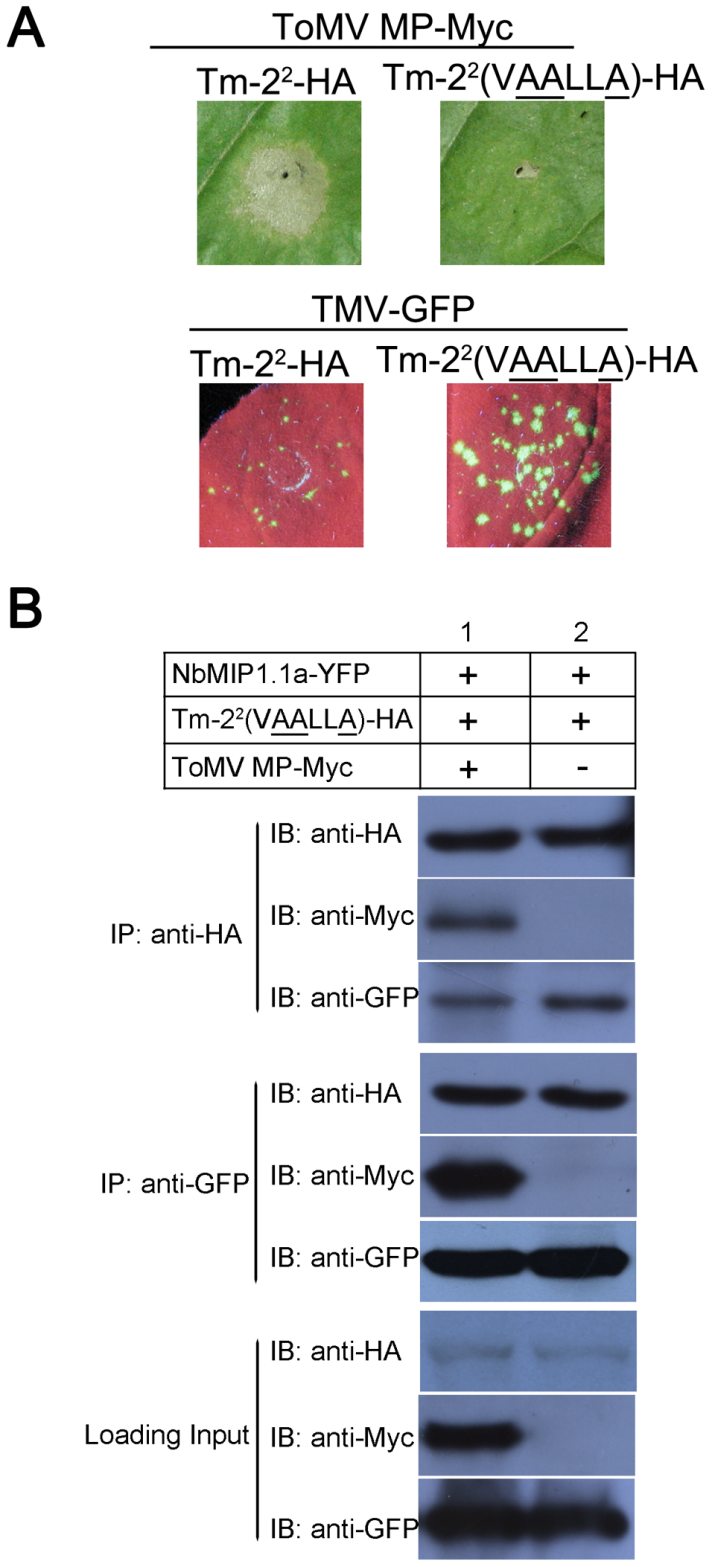
Tm-2^2^ (VAALLA) mutant, NbMIP1.1a and ToMV MP could exist in the same complex in plants. (**A**) The Tm-2^2^ (VAALLA) mutant did not induce HR when co-expressed with ToMV MP-Myc (upper), and failed to induce resistance against TMV-GFP (lower). Tm-2^2^-HA or Tm-2^2^ (VAALLA)-HA were agroinfiltrated into wild-type *N. benthamiana* leaves with either ToMV MP-Myc (upper) or TMV-GFP (lower). Photos were taken at 4 dpi under UV light. (**B**) Tm-2^2^ (VAALLA)-HA co-immunoprecipitated with both ToMV MP and NbMIP1.1a (top). YFP-NbMIP1.1a co-immunoprecipitated with both ToMV MP and Tm-2^2^ (VAALLA) mutant (middle). Loading controls were also analyzed by western blot (bottom). Tm-2^2^ (VAALLA)-HA and YFP-NbMIP1.1a were co-expressed with ToMV MP-Myc or empty Myc vector in *N. benthamiana* leaves through agroinfiltration. At 60 hpi, leaf lysates were immunoprecipitated with anti-HA or anti-GFP beads, then the immunoprecipitates were assessed by western blotting using anti-HA, anti-Myc or anti-GFP antibodies as indicated.

Since NbMIP1s interact with both Tm-2^2^ and its corresponding Avr protein ToMV MP, we next used co-IP assays to test whether Tm-2^2^, NbMIP1s and ToMV MP exist in same complex. We co-expressed YFP-NbMIP1.1a, Tm-2^2^-HA and ToMV MP-Myc in *N. benthamiana*. As expected, HR cell death was induced by co-expression of Tm-2^2^-HA and ToMV MP-Myc (along with YFP-NbMIP1.1a). Therefore, we collected leaf tissue for co-IP assays before visible cell death, but we did not detect any Tm-2^2^-HA protein (data not shown), perhaps due to extremely low expression of Tm-2^2^ during HR. Alternatively, we used an HA-tagged Tm-2^2^ mutant that does not induce HR and is not involved in resistance activation and viral recognition. To identify this mutant, we also examined other NBS-LRR proteins besides Tm-2^2^. The Tm-2^2^ LRR domain interacts with NbMIP1s and has been reported to be response for the recognition of ToMV MP [28], [32]. A mutation in the NB domain of L6 does not interact with AvrL567 proteins in yeast [33], and a mutation in the NB domain of N that prevents its activation also abolishes N's association with SPL6 [34], suggesting that NB domains could be involved in R protein activation and recognition. Therefore, we used a mutation in the Tm-2^2^ CC domain to prevent the induction of HR for co-IP assays. We chose the Tm-2^2^ EDVID mutant because the highly conserved EDVID motif in the CC domain is supposed to be important for receptor post-activation signaling [35], [36], [37] but not for Avr recognition. Therefore, we used the HA-tagged Tm-2^2^ EDVID mutant (Tm-2^2^ (VAALLA)-HA) to replace Tm-2^2^-HA for co-expression with YFP-NbMIP1.1a and ToMV MP-Myc. Tm-2^2^ (VAALLA) did not induce an HR when coexpressed with ToMV MP-Myc (Figure 3A), consistent with the idea that the conserved EDVID motif in the CC domain is important for the function of CC-NB-LRR resistance proteins [35]. Co-IP assays showed that YFP-NbMIP1.1a co-immunoprecipitated with both ToMV MP-myc and Tm-2^2^ (VAALLA)-HA, and Tm-2^2^ (VAALLA)-HA co-immunoprecipitated with both ToMV MP-Myc and YFP-NbMIP1.1a (Figure 3B). These results suggest that NbMIP1.1a, Tm-2^2^ and ToMV MP may interact in the same complex.

### Subcellular Localization of NbMIP1s

**Figure 4:**
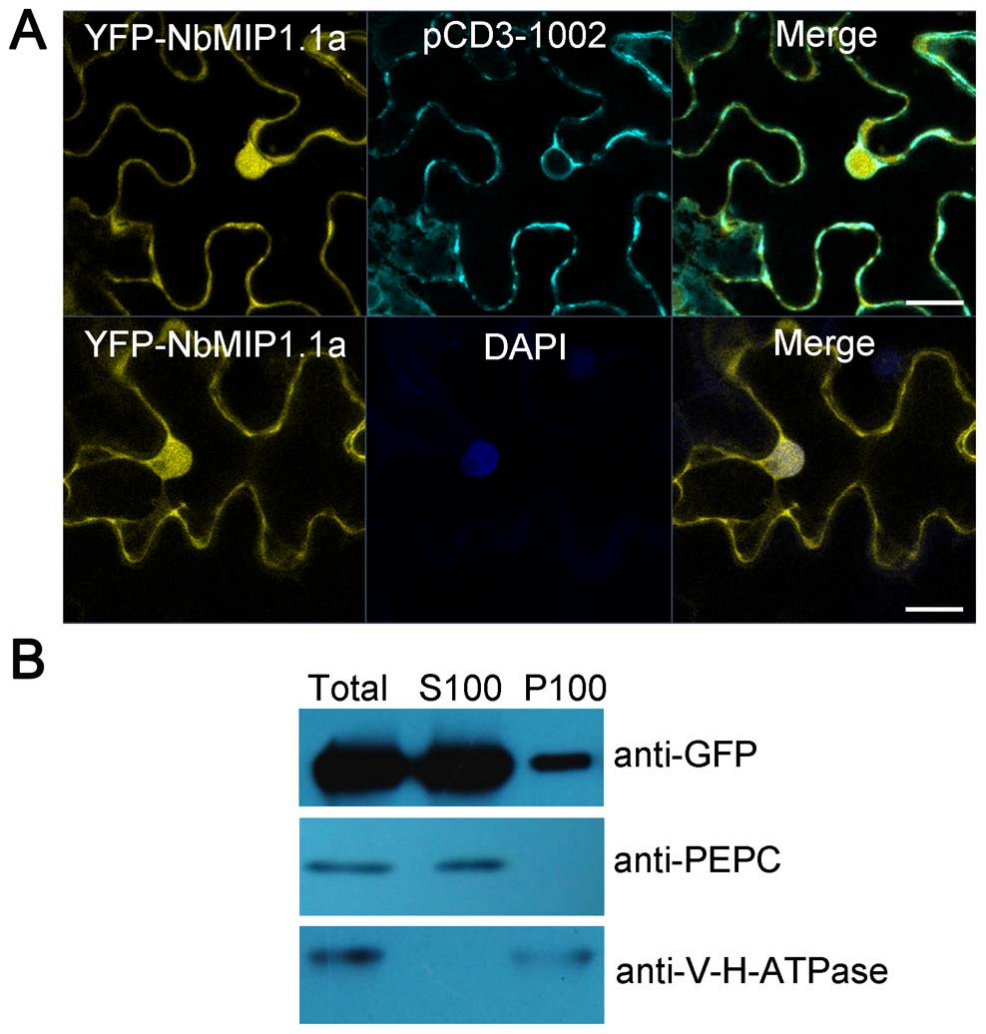
The subcellular localization of NbMIP1.1a in *N. benthamiana* cells. (**A**) Confocal image of the subcellular localization of NbMIP1.1a in leaf epidermal cells. YFP-NbMIP1.1a was transiently expressed in leaves of *N. benthamiana* via agroinfiltration and imaged at 48 hpi using a Zeiss LSM 710 laser scanning microscope. YFP signal revealed that NbMIP1.1a is present in the cell membrane, cytoplasm and nucleus. PCD3-1002: a CFP-tagged plasma membrane marker [81]. DAPI: staining for nuclei. Scale bar represents 20 µm. (**B**) YFP-NbMIP1.1a was found in both the soluble fraction and the membrane fraction (upper panel). Protein extracts were centrifuged at 100,000×g to produce crude soluble (S100) and microsomal (P100) fractions. Fractions were analyzed by western blot following separation by SDS-PAGE. The gels were probed using anti-GFP, anti-V-H-ATPase (vacuolar H-ATPase subunit, a vacuolar membrane marker) and anti-PEPC (phosphoenolpyruvate carboxylase, a cytosolic marker) antibodies as indicated.

To determine the subcellular localization of NbMIP1s *in planta*, we generated NbMIP1s tagged with yellow fluorescent protein (YFP) reporter at their N- or C-terminus (e.g., YFP-NbMIP1.1a or NbMIP1.1a-YFP) under the control of the CaMV 35S promoter and transiently expressed them in *N. benthamiana* plants using agroinfiltration. We examined YFP localization in the infiltrated leaves by confocal microscopy. Both YFP-NbMIP1.1a and NbMIP1.1a-YFP were detected perhaps at the cell membrane and in the cytoplasm and nucleus (Figure 4A; Figure S6A). Similarly, confocal microscopy of YFP-tagged versions of the other NbMIP1s showed that the other NbMIP1s are also localized at the cell membrane and in the cytoplasm and nucleus (Figure S7).

We also confirmed these observations by subcellular fractionation. To this end, total protein was extracted from leaves expressing YFP-NbMIP1.1a or NbMIP1.1a-YFP and the membrane fraction (P100) and the soluble fraction (S100) were isolated as described previously [38]. Western blot assays with anti-GFP antibodies indicated that both YFP-NbMIP1.1a and NbMIP1.1a-YFP were in the soluble and microsomal fractions (Figure 4B; Figure S6B). These results showed that NbMIP1.1a is localized at cell membrane and in the cytoplasm and nucleus, similar to the subcellular localization of its Arabidopsis homologue AtJ3 [16]. We tested the subcellular localization of other YFP-tagged NbMIP1s and found that other NbMIP1s are also localized at cell membrane and in the cytoplasm and nucleus (Figure S7). Furthermore, we did not find any change in the subcellular localization of NbMIP1.1a during *Tm2^2^*-mediated resistance (Figure S8).

### Expression of *NbMIP1s* during TMV Infection and Resistance

To investigate the potential involvement of *NbMIP1s* in virus-plant interactions, we examined their expression during TMV infection. Total RNAs were extracted from GFP-tagged TMV (TMV-GFP) infected *N. benthamiana* local leaves at 0, 2, 4, 6 days after TMV-GFP infection, and used for analysis of the expression of *NbMIP1s*. Real-time RT-PCR showed that mRNA levels of all *NbMIP1s*, but not *NbMIP1L1*, increased during TMV infection (Figure S9).

We also examined the expression of *NbMIP1s* during *Tm-2^2^*-mediated resistance against TMV. This experiment was performed in transgenic *Tm-2^2^ N. benthamiana* line TM#1, in which *Tm-2^2^* confers extreme resistance against TMV [39] and ToMV (Figure S10). In these *Tm-2^2^* transgenic plants, TMV MP, but not the 50KD helicase domain of TMV replicase (TMV P50), induced an HR (Figure S11), suggesting that, as for ToMV, *Tm-2^2^* confers resistance to TMV by recognizing viral MP. We extracted total RNA from TMV-GFP infected TM#1 local leaves at 0, 8, 16, 24, 48 hours after infection. Real-time RT-PCR showed that mRNA levels of all *NbMIP1s*, but not *NbMIP1L1*, increased during *Tm-2^2^*-mediated TMV resistance (Figure S12).

### Silencing of *NbMIP1s* Compromises *Tm-2^2^*-Mediated Resistance to TMV and ToMV

**Figure 5:**
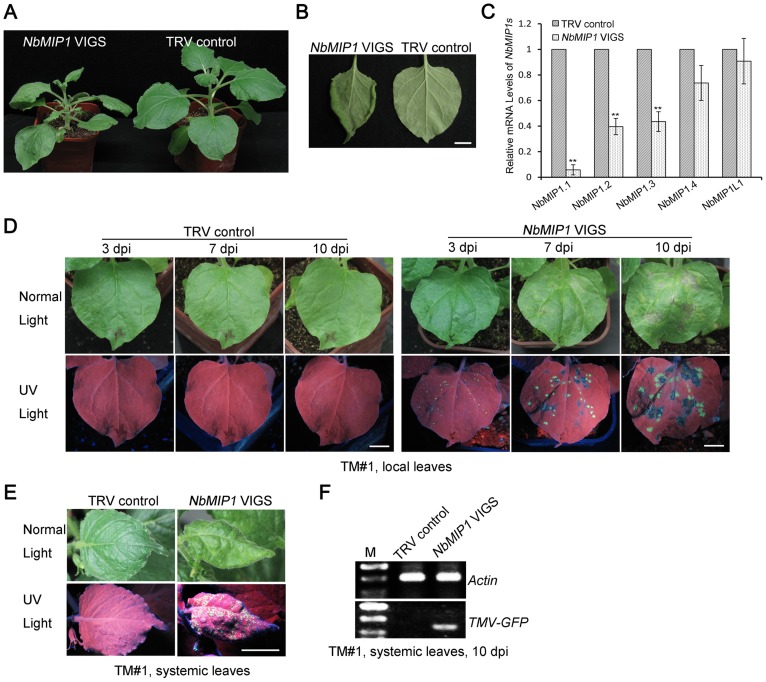
*Tm-2^2^*-mediated resistance against TMV requires *NbMIP1s*. (**A**) Phenotype of *NbMIP1s*-silenced and TRV control *N. benthamiana* plants. *NbMIP1s*-silenced *N. benthamiana* plants developed dwarfed stems and crinkled leaves compared to TRV-infected control plants. (**B**) Leaves of *NbMIP1*-silenced and TRV control *N. benthamiana* plants. The leaf edge of *NbMIP1s*-silenced plants curled downward but TRV-infected control leaves looked normal (right side). Photos were taken at 14 days post agroinfiltration for VIGS. (**C**) Real time RT-PCR to confirm the suppression of *NbMIP1s*, and the *Actin* mRNA levels were used as internal controls. *NbMIP1* VIGS: silencing using pTRV2-*NbMIP1*. (**D**) Silencing of *NbMIP1s* caused the appearance of TMV-GFP infection foci and visible HR lesions in the inoculated leaves of *NbMIP1s*-silenced *Tm-2^2^*-containing TM#1 plants. (**E**) Silencing of *NbMIP1s* compromised *Tm-2^2^*-mediated resistance against TMV, and TMV-GFP spread from the inoculated leaves into the upper non-inoculated leaves of *NbMIP1s*-silenced TM#1 plants. TRV-infected TM#1 plants were used as negative controls. Photos were taken at 10 days post TMV-GFP infection (dpi). Scale bars represent 1 cm. (**F**) RT-PCR was performed to confirm the presence of TMV-GFP in systemic leaves of *NbMIP1s*-silenced TM#1 plants.

To investigate the biological role of *NbMIP1s* in plants, we used a *Tobacco rattle virus* (TRV)-based VIGS system [40] to silence *NbMIP1s*. We first investigated the role of *NbMIP1s* in *Tm-2^2^*-mediated resistance against TMV infection. As expected, the non-silenced TM#1 control plants were extremely resistant to TMV infection and neither HR and nor TMV-GFP infection foci were observed in the inoculated leaves (Figure S13B). Similarly, silencing of *NbMIP1s* using their gene-specific 3′-UTR had no effect on *Tm-2^2^*-mediated resistance against TMV infection; these silenced plants still showed extreme resistance to TMV-GFP (Figure S13B). Real-time RT-PCR showed that silencing of individual *NbMIP1s* greatly reduces the mRNA levels of the corresponding *NbMIP1*, but has no significant effect on mRNA levels of the other *NbMIP1* homologs (Figure S13A), suggesting that silencing of individual *NbMIP1s* is effective and specific. Considering that multiple NbMIP1s interact with Tm-2^2^, we speculated that the *NbMIP1s* have redundant functions. *NbMIP1s* share high sequence identity in their coding DNA sequences; therefore, we tried to silence multiple *NbMIP1s* using these regions of sequence identity. For this purpose, we cloned the *NbMIP1.1a* coding sequence into pTRV2 to generate pTRV2-NbMIP1. Silencing of *NbMIP1s* using pTRV2-NbMIP1 induced developmental abnormalities including dwarf and downward curl leaf compared to pTRV2-infected control plants (Figure 5A–5B). Real-time RT-PCR analysis using *Actin* as an internal reference indicated that pTRV2-NbMIP1 infection reduced total mRNA levels of *NbMIP1.1* by approximately 90% and mRNA levels of *NbMIP1.2* and *NbMIP1.3* by about 60%, but had no significant effect on mRNA levels of *NbMIP1.4* and *NbMIP1L1* (Figure 5C).

We next tested whether the silenced TM#1 plants showed any effects on resistance to TMV or ToMV. We infected the *NbMIP1s*-silenced TM#1 plants with TMV-GFP and observed TMV-GFP infection foci at 3 days post TMV infection (dpi) and subsequent development of severe visible necrotic local lesions (Figure 5D, right). Moreover, at 10 dpi TMV-GFP was able to spread into the upper leaves of *NbMIP1*-silenced TM#1 plants but not into the upper leaves of the non-silenced control TM#1 plants (Figure 5E). TMV RNA was readily detected by RT-PCR in the systemic leaves of *NbMIP1*-silenced TM#1 plants but not in the leaves of non-silenced control TM#1 plants (Figure 5F). In addition, we found ToMV induced local (Figure S14A) and systemic (Figure S14B) necrosis in the *NbMIP1s*-silenced TM#1 plants but not in the non-silenced control TM#1 plants, suggesting that ToMV spreads into the upper non-inoculated leaves of the *NbMIP1*-silenced TM#1 plants. Indeed, we detected ToMV by RT-PCR in the systemic leaves of *NbMIP1s*-silenced but not in the leaves of non-silenced TM#1 plants (Figure S14C).

We also performed VIGS using the coding sequences of other *NbMIP1s*. Silencing of *NbMIP1s* using the coding sequence of *NbMIP1.2*, *NbMIP1.3* or *NbMIP1.4a* reduced expression of multiple *NbMIP1* genes (Figure S15A) and caused some leaf developmental defects (Figure S15B), as we observed for silencing using the *NbMIP1.1a* coding sequence. Silencing with the coding sequences of *NbMIP1.2*, *NbMIP1.3* or *NbMIP1.4b*, but not *NbMIP1L1*, caused the loss of *Tm-2^2^*-mediated resistance (Figure S15C–15F). However, silencing using the coding sequence of *NbMIP1.4b* only caused the loss of *Tm-2^2^*-mediated local resistance, consistent with the observation that it reduced the expression of fewer *NbMIP1* genes. These results demonstrate that *Tm-2^2^*-mediated resistance requires *NbMIP1s*.

We also confirmed the effect of *NbMIP1s* on *Tm-2^2^*-mediated resistance by transiently expressing *NbMIP1* hairpin RNAi construct with TMV-GFP using agroinfiltration. Plants that agroinfiltrated with the *NbMIP1.1a* hairpin RNAi construct alone had reduced mRNA levels of *NbMIP1.1* (50%), *NbMIP1.2* (62%) and *NbMIP1.3* (65%) compared to vector control plants, as measured by real-time RT-PCR (Figure S16A). Co-infiltration of TMV-GFP with the *NbMIP1* RNAi construct, but not with empty pRNAi-LIC vector, resulted in TMV-GFP spreading into the upper leaves of *NbMIP1* RNAi TM#1 plants (Figure S16C). Furthermore, TMV RNA was detected by RT-PCR in the systemic leaves of *NbMIP1* RNAi TM#1 plants but not in those of control TM#1 plants (Figure S16D). Interestingly, plants agroinfiltrated with *NbMIP1* hairpin RNAi construct alone did not show any visible developmental phenotype (Figure S16B), perhaps due to the reduced silencing efficiency compared to VIGS. These results suggest that the loss of *Tm-2^2^*-mediated resistance caused by silencing of *NbMIP1s* is not due to plant developmental abnormalities.

Taken together, the above results suggest that multiple NbMIP1s are required for *Tm-2^2^*-mediated resistance.

### Suppression of *NbMIP1s* Reduces TMV Infection

**Figure 6:**
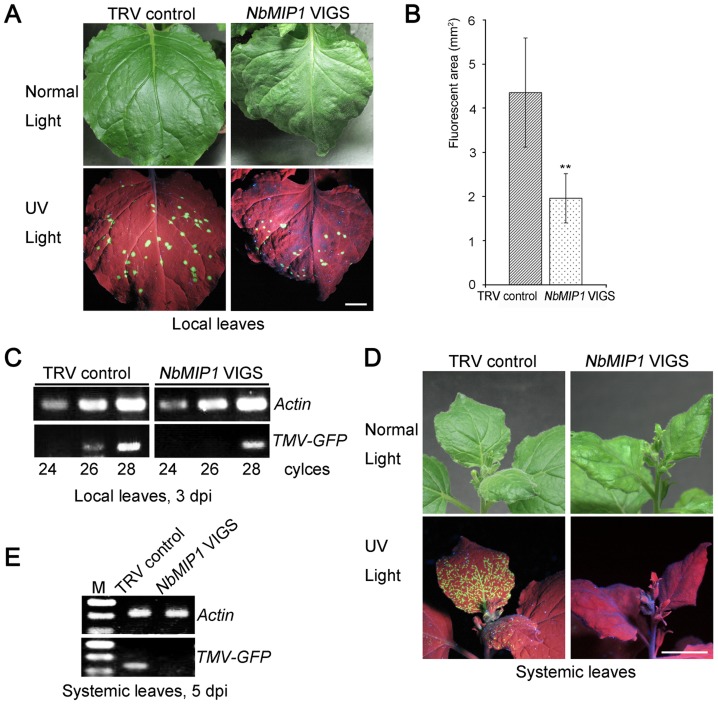
*NbMIP1s* are involved in TMV movement. (**A**) Silencing of *NbMIP1s* reduced cell-to-cell movement of TMV. TMV-GFP formed much smaller infection foci in *NbMIP1s*-silenced plants (right) compared to control plants (left). Photos were taken at 3 dpi. Scale bars represent 1 cm. (**B**) Average sizes of TMV-GFP foci at 3 dpi are shown. All values in bar graphs represent means with standard deviation. **: p<0.01 (Student's *t*-test). Data are from 3 independent experiments, and 9 leaves for each construct per experiment. (**C**) RT-PCR to confirm that the suppression of *NbMIP1s* reduced TMV-GFP vRNA levels in local inoculated leaves at 3 dpi. (**D**) Silencing of *NbMIP1s* delayed systemic TMV movement. At 5 dpi, TMV-GFP had already spread into the upper non-inoculated leaves in control plants (left), but not into the systemic leaves in *NbMIP1s*-silenced plants (right). Scale bars represent 1 cm. (**E**) RT-PCR to confirm the delay of TMV-GFP systemic movement in *NbMIP1s*-silenced plants at 5 dpi. For each RT-PCR, *Actin* was used as an internal control.

Since NbMIP1s interact with ToMV MP, which is responsible for viral cell-to-cell movement, we tested the role of *NbMIP1s* in TMV infection. Silencing of *NbMIP1s* using their gene-specific 3′-UTRs had no effect on TMV infection (Figure S17). Thus, we performed VIGS using pTRV2-NbMIP1 to silence multiple *NbMIP1s* in wild-type *N. benthamiana* plants, which do not have *Tm-2^2^*, and then infected these plants with TMV-GFP. At 3 dpi, we found that the size of single TMV-GFP infection foci was significantly smaller in the inoculated leaves of *NbMIP1*-silenced plants than non-silenced control plants (Figure 6A and 6B), suggesting that silencing of *NbMIP1* caused slower TMV-GFP cell-to-cell movement. In addition, TMV RNA levels were reduced in the *NbMIP1*-silenced plants compared to the non-silenced control plants (Figure 6C). At 5 dpi, TMV-GFP was able to spread into the upper non-inoculated leaves of the control plants but not into that of *NbMIP1*-silenced plants (Figure 6D). Consistent with these observations, no TMV RNA was detected in the systemic leaves of *NbMIP1*-silenced plants (Figure 6E). However, TMV-GFP was able to spread into the upper non-inoculated leaves in *NbMIP1*-silenced plants 7 days post virus infection (data not shown), suggesting that suppression of *NbMIP1* delayed TMV systemic movement.

We also tested the effect of silencing of *NbMIP1s* on TMV replication in *N. benthamiana* mesophyll protoplasts. We prepared protoplasts from the leaves of *NbMIP1*-silenced and TRV control plants, and then transfected them with TMV-GFP RNA. We extracted total RNAs at 0, 24, 48, 72 hours post transfection, and measured TMV RNA levels by real time RT-PCR using *Actin* as an internal control. Real time RT-PCR analysis showed silencing of *NbMIP1s* had no significant effect on the TMV-GFP genomic and TMV-GFP CP RNA levels although both increased dramatically during the first 24 hours post transfection (Figure S18), suggesting that *NbMIP1s* are not essential for TMV replication. Taken together, these data suggest that *NbMIP1s* are required for TMV movement, but not for TMV replication.

### NbMIP1s Are Required for ToMV MP and Tm-2^2^ Protein Stability

**Figure 7:**
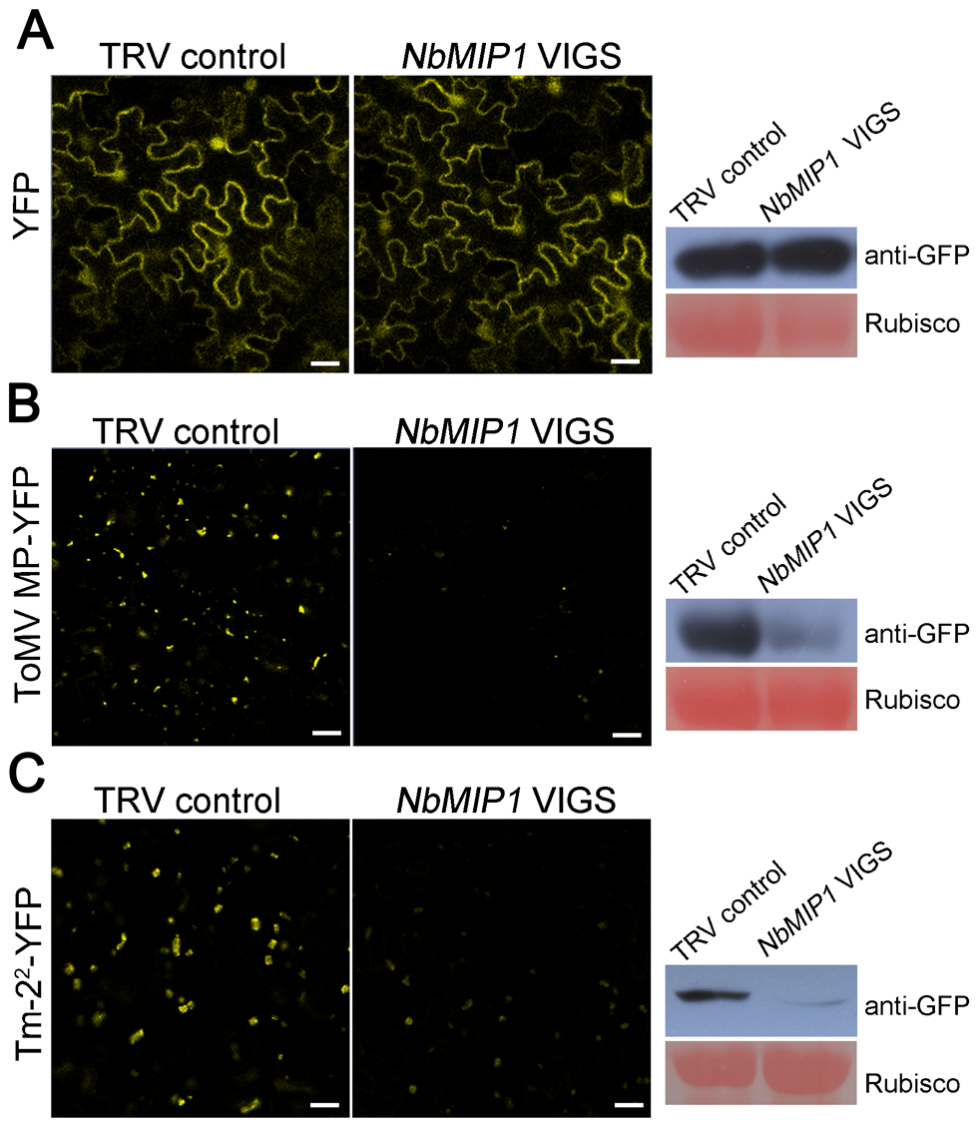
NbMIP1s are essential for the stability of ToMV MP and Tm-2^2^ protein. Effect of silencing of *NbMIP1s* on the steady-state levels of YFP alone (**A**), ToMV MP-YFP (**B**) and Tm-2^2^-YFP (**C**). Silencing of *NbMIP1s* reduced the fluorescence intensity of ToMV MP-YFP (B, left) and Tm-2^2^-YFP (C, left) but not that of YFP alone (A, left). YFP, ToMV MP-YFP and Tm-2^2^-YFP T-DNA constructs were agroinfiltrated into leaves of *NbMIP1s* silenced plants and TRV control plants respectively and confocal images (left panels) were taken at 72 hpi. Scale bars represent 20 µm. Proteins were extracted from the agro-infiltrated leaf areas and analyzed by SDS-PAGE, followed by western blot with anti-GFP antibody (right panels). Western blot assays further showed that silencing of *NbMIP1s* specifically reduced the protein level of ToMV MP-YFP (B, right) and Tm-2^2^-YFP (C, right) but had no effect on YFP alone (A, right). Ponceau Red staining of Rubisco indicates equal loading (lower panel). All experiments were performed three times with three replicated samples in each experiment.

To investigate the possible role of *NbMIP1s* in protein stability of ToMV MP and Tm-2^2^, we transiently expressed C-terminal YFP-tagged ToMV MP (ToMV MP-YFP) and C-terminal YFP-tagged Tm-2^2^ (Tm-2^2^-YFP) under the control of the CaMV 35S promoter in wild type *N. benthamiana* plants. ToMV MP-YFP induces an HR cell death when co-expressed with *Tm-2^2^* and Tm-2^2^-YFP induces an HR cell death when co-expressed with ToMV *MP* (Figure S19), indicating that the ToMV MP-YFP and Tm-2^2^-YFP protein fusions are functional. We then silenced multiple *NbMIP1s* using pTRV2-NbMIP1 in wild-type *N. benthamiana* plants. Silencing of *NbMIP1s* had no effect on fluorescence intensity of YFP alone (Figure 7A, left). However, the fluorescence intensity of ToMV MP-YFP and Tm-2^2^-YFP were greatly reduced in *NbMIP1*-silenced plants compared to non-silenced control plants (Figure 7B and 7C, left). We confirmed this apparent change by western blot assays using anti-GFP antibodies, which showed that silencing of *NbMIP1* resulted in a substantial reduction of the steady-state levels of ToMV MP-YFP and Tm-2^2^-YFP (Figure 7B and 7C, right), but had no effect on the protein level of YFP alone (Figure 7A, right). We obtained similar results using transgenic plants expressing Myc-tagged ToMV MP (ToMV MP-Myc), as silencing of *NbMIP1s* reduced the protein level of ToMV MP-Myc (Figure S20A). In addition, real-time RT-PCR analysis showed that suppression of *NbMIP1s* had no effect on mRNA levels of *MP-Myc*, *MP-YFP* and *Tm-2^2^-YFP* (Figure S20B). These results suggest that NbMIP1s are required for the protein stability of both ToMV MP and Tm-2^2^.

### NbSGT1 Interacts with Both NbMIP1.1a and Tm-2^2^ and Is Required for *Tm-2^2^*-Mediated Resistance to TMV

**Figure 8:**
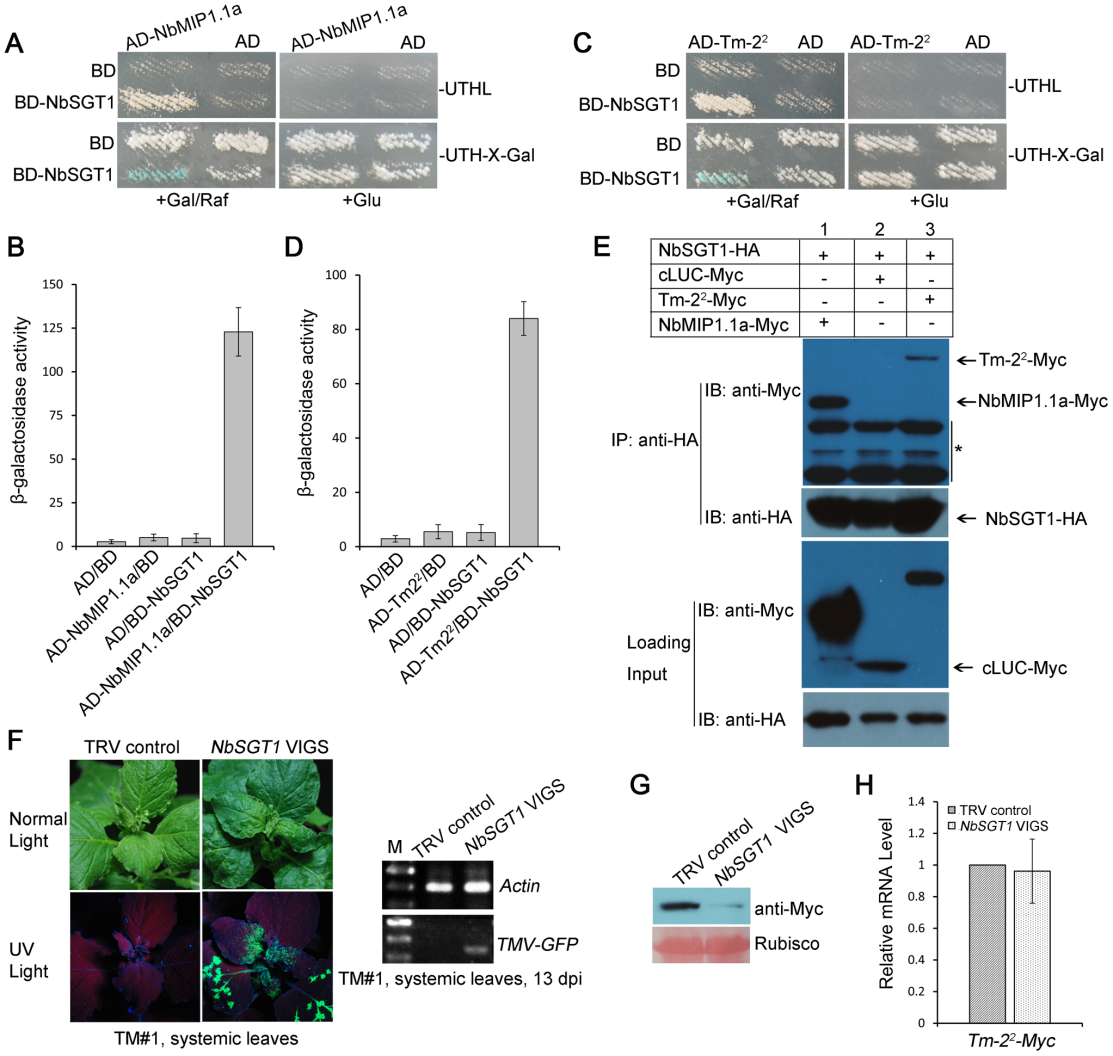
NbSGT1 interacts with NbMIP1.1a and Tm-2^2^ and is required for *Tm-2^2^*-mediated resistance to TMV. (**A–D**) NbSGT1 interacts with both NbMIP1.1a and Tm-2^2^ in yeast. (A, C) Yeast cells harboring AD-NbMIP1.1a (A), AD-Tm-2^2^ (C) transformed with AD-NbSGT1 grew on Leu^-^ selection medium and turned blue on X-gal medium plus Gal/Raf but not on medium plus glucose. Yeast cells transformed with either BD or AD vector alone for control assays showed no growth on Leu^−^ selection medium and remained white on X-gal medium containing either Gal/Raf or glucose. For each experiment, yeast strains were maintained at 28°C for 5 days. (B, D) Quantification of β-galactosidase activities in yeast two-hybrid interactions. (**E**) NbSGT1 co-immunoprecipitated (co-IP) with NbMIP1.1a and Tm-2^2^. NbSGT1-HA was co-expressed with NbMIP1.1a-Myc or Tm-2^2^-Myc respectively in *N. benthamiana* leaves through agroinfiltration. Coexpression of NbSGT1-HA and cLUC-Myc was used as a negative control. At 2 dpi, leaf lysates were immunoprecipitated with anti-HA beads, then the immunoprecipitates were assessed by western blotting using anti-Myc (upper panel) and anti-HA antibodies (middle panel). In addition to immunoblotting for co-IP, presence of NbSGT1-HA, NbMIP1.1a-Myc, Tm-2^2^-Myc and cLUC-Myc in the cell lysates were also analyzed (lower panel). * indicates nonspecific bands. (**F**) Silencing of *NbSGT1* in *Tm-2^2^* transgenic TM#1 plants caused TMV-GFP spreading into the upper non-inoculated leaves (left). TRV-infected TM#1 plants were used as negative controls. Photos were taken at 10 dpi. RT-PCR was performed to confirm the presence of TMV-GFP in systemic leaves of *NbSGT1*-silenced TM#1 plants (right). (**G**) Silencing of *NbSGT1* reduced the protein level of Tm-2^2^-Myc. Ponceau Red staining of Rubisco indicates equal loading (lower panel). Experiments were performed three times with three replicated samples in each experiment. (**H**) Real-time RT-PCR showed that silencing of *NbSGT1* had no effect on the expression level of *Tm-2^2^-Myc*, and *Actin* mRNA levels were used as the internal control. Data are shown as means ± SD for 3 independent triplicate experiments (Student's *t*-test).

Since we identified SGT1 in yeast two-hybrid screen using Tm-2^2^-LRR as bait, we tested the interaction between SGT1 and NbMIP1 or Tm-2^2^. First, we expressed NbMIP1.1a and Tm-2^2^ as fusions to the B42 activation domain (AD) (AD-NbMIP1.1a, AD-Tm-2^2^), and NbSGT1 [29], [30] as a fusion to the LexA DNA binding domain (BD) (BD-NbSGT1). Yeast transformed with BD-NbSGT1 and either AD-NbMIP1.1a or AD-Tm-2^2^ grew on Leu^−^ selection media, turned blue on X-gal plates containing Gal/Raf but not glucose and showed significant levels of β-galactosidase activity (Figure 8A–8D). Control yeast containing BD or AD alone was not able to grow on selection plates or turn X-gal blue on glucose or Gal/Raf (Figure 8A–8D). These results suggest that NbSGT1 interacts with both NbMIP1.1a and Tm-2^2^ in yeast.

To test whether NbSGT1 intereacts with NbMIP1.1a or Tm-2^2^ *in planta*, we performed Co-IP assays. HA-tagged NbSGT1 (NbSGT1-HA) was transiently co-expressed with a Myc-tagged NbMIP1.1a (NbMIP1.1a-Myc) or Myc-tagged Tm-2^2^ (Tm-2^2^-Myc) under the control of the CaMV 35S promoter in *N. benthamiana*. Total proteins extracted from leaves co-infiltrated with *Agrobacterium* carrying the NbSGT1-HA expression cassette and *Agrobacterium* carrying either the control cLUC-Myc, NbMIP1.1a-Myc or Tm-2^2^-Myc expression cassettes were immuno-precipitated using anti-HA antibodies. The resulting precipitates were analyzed by western blot using anti-Myc antibodies. We observed that NbSGT1 co-immunoprecipitated with either NbMIP1.1a or Tm-2^2^, but not with cLUC-Myc (Figure 8E). These results demonstrate that NbSGT1 interacts with NbMIP1.1a and Tm-2^2^ in plants.

Furthermore, we confirmed the NbSGT1 association with NbMIP1.1a, and tested the NbSGT1 interaction with other NbMIP1s *in planta* by LCI assays. We found that NbSGT1 interacts with all 6 NbMIP1s (NbMIP1.1a, NbMIP1.1b, NbMIP1.2, NbMIP1.3, NbMIP1.4a, and NbMIP1.4b), but not with the cLUC control. In addition, NbSGT1 also interacted weakly with NbMIP1L1 *in planta* (Figure S21). These results suggested that NbSGT1 interacts with NbMIP1s in plants.

Since NbSGT1 interacts with both NbMIP1s and Tm-2^2^, we used TRV VIGS to silence *NbSGT1* [29] to investigate its role in *Tm-2^2^*-mediated resistance against TMV. Silencing of *NbSGT1* allowed TMV-GFP to spread into the upper non-inoculated leaves of *Tm-2^2^* transgenic *N. benthamiana* TM#1 plants at 13 dpi (Figure 8F), suggesting that *NbSGT1* is required for *Tm-2^2^*-mediated resistance. Furthermore, we also tested the effect of silencing of *NbSGT1* on protein and RNA levels by transiently expressing *Tm-2^2^* as a C-terminal Myc-tagged fusion protein (Tm-2^2^-Myc) under the control of CaMV 35S promoter in wildtype *N. benthamiana* plants. Western blot assays using anti-Myc antibodies showed that silencing *NbSGT1* affected steady-state Tm-2^2^ protein levels (Figure 8G); however real time RT-PCR showed that silencing *NbSGT1* did not affect *Tm-2^2^* mRNA levels (Figure 8H). These results suggest that NbSGT1 is required for Tm-2^2^ protein stability.

### *NbMIP1s* Are Required for Plant Immunity Mediated by Other *R* Genes and General Elicitors

**Figure 9:**
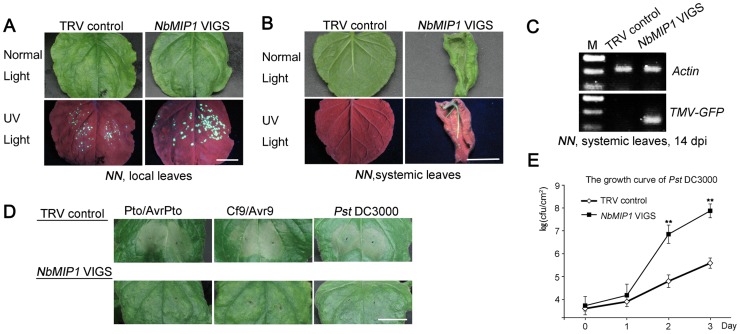
NbMIP1s are required for plant immunity mediated by multiple *R* genes and general elicitors. (**A**) Silencing of *NbMIP1s* resulted in larger TMV-GFP infection foci in transgenic *N*-containing *N. benthamiana* plants (*NN*) plants. Photos were taken under normal or UV light at 7 dpi. (**B**) Silencing of *NbMIP1s* compromised *N*-mediated resistance to TMV-GFP in *NN* plants and TMV-GFP spread into the upper non-inoculated leaves at 14 dpi. (**C**) RT-PCR to confirm the presence of TMV-GFP RNA in the upper, non-inoculated leaves of *NbMIP1s*-silenced *NN* plants. (**D**) Silencing of *NbMIP1s* delayed HR mediated by several *R* genes and nonhost *Pseudomonas syringae pv. tomato* DC3000 (*Pst* DC3000). All R-Avr pairs were expressed in wild type plants. Transient co-expression of Pto with avrPto, or Cf9 with avr9 was performed using agroinfiltration and *Pst* DC3000 was inoculated in *NbMIP1s*-silenced and non-silenced control plants. The leaf images of *Pst* DC3000 were taken at 12 hpi, and the other images corresponding to R-Avr pairs were taken at 3 dpi. Scale bars represent 1 cm. (**E**) Silencing of *NbMIP1s* compromised nonhost resistance against *Pst* DC3000 and caused more bacterial growth. Bacterial growth was monitored at 0, 1, 2 and 3 dpi. Each data point represents the mean ± SEM of 3 replicate samples (** p<0.01, Student's *t*-test). All experiments were performed at least three times using three or more plants in each experiment.

Since SGT1 is required for resistance mediated by many Resistance (R) proteins [30], [41], [42], [43], [44], [45], [46], [47] and interacts with NbMIP1s, we hypothesized that *NbMIP1s* may have a role in plant immunity mediated by other *R* genes as well as general elicitors. First, we tested the role of *NbMIP1s* in *N*-mediated resistance to TMV in *N*-containing *N. benthamiana* (*NN*) plants. Indeed, we observed larger single TMV-GFP infection foci in the inoculated leaves of *NN* plants silenced for *NbMIP1* with pTRV2-NbMIP1 (Figure 9A). In addition, TMV-GFP was able to spread into the upper non-inoculated leaves of *NbMIP1*-silenced *NN* plants but not into the systemic leaves of the non-silenced control *NN* plants at 14 dpi (Figure 9B, 9C).

Second, we tested the role of *NbMIP1s* in HR development mediated by other *R* genes. To this end, we co-expressed the bacterial resistance gene *Pto* and its corresponding *Avr* gene, *AvrPto* [48] and also co-expressed the fungal resistance gene *Cf9* and its corresponding *Avr* gene, *Avr9* [49]. We also tested the effect of *NbMIP1* silencing on nonhost resistance by infecting plants with *Pst* DC3000. We observed that the HR induced by these treatments was delayed in the *NbMIP1*-silenced plants compared to the non-silenced control plants (Figure 9D). Furthermore, silencing of *NbMIP1* compromised nonhost resistance to *Pst* DC3000 and resulted in increased growth of *Pst* DC3000 (Figure 9E). Together, these results demonstrate that *R* gene mediated resistance and nonhost resistance require *NbMIP1s*.

## Discussion

In this study, we found that NbMIP1s, a group of J-domain proteins, interact with the viral effector ToMV MP, with its corresponding R protein Tm-2^2^ and with the resistance signaling component SGT1. Silencing of *NbMIP1s* compromised not only TMV infection but also plant immunity induced by several *R* genes and a nonhost pathogen. Furthermore, we showed that NbMIP1s are necessary for maintaining protein stability of both MP and Tm-2^2^. These results suggest that *NbMIP1s* are required not only for virus infection but also for plant immunity, and act by maintaining protein stability of viral effector and R proteins.

One potential limitation of the *in vivo* interaction data of NbMIP1s with other proteins (ToMV MP, Tm-2^2^ and NbSGT1) is that these proteins were overexpressed. However, both NbMIP1 and NbSGT1 did not interact with the negative control proteins (nLUC or cLUC), and expression of *Tm-2^2^* driven by CaMV 35S promoter confers virus resistance in tobacco plants similar to *Tm-2^2^* in its native system [13], [39]. Furthermore, ToMV MP with a C-terminal tag and Tm-2^2^ with a C-terminal tag are functional to induce HR when co-expressed with wildtype *Tm-2^2^* and *ToMV MP*. Therefore, we believe these data to be biologically significant.

Another potential limitation is that we examined *Tm-2^2^*-mediated resistance in *N. benthamiana*, not in its native tomato. However, *Tm-2^2^* mediated resistance in tobacco and the specific recognition between *Tm-2^2^* and the Tobamovirus MPs remained the same in transgenic tobacco plants as in its native tomato host ([13], [39]; Figure S10–S11), suggesting that transgenic *Tm-2^2^ Nicotiana benthamiana* plants can be used to study *Tm-2^2^*-mediated resistance.

### Role of NbMIP1s in Virus Infection

J-domain proteins interact with capsid proteins of *Potato Virus Y* and *Potato virus A*, and are required for Potato virus Y infection [50]. Recently, GmHSP40.1, a J-domain protein, is reported to negatively regulate virus infection and its silencing enhanced the susceptibility of soybean plants to *Soybean mosaic virus* [27]. It is possible that different DnaJ proteins play distinct roles in various processes.

MPs of TMV and ToMV are responsible for viral cell-to-cell movement by increasing the plant plasmodesmal size-exclusion limit during viral infection. Many host factors have been identified to cooperate with TMV/ToMV MP during this process [51], [52], [53]. For example, the tobacco J-domain protein MPIP1 is required for TMV cell-to-cell movement [52]. In this study, we identified a group of novel J-domain proteins, the NbMIP1s, which interact with ToMV/TMV MP. Silencing of *NbMIP1s* reduced TMV cell-to-cell and systemic movement but not TMV replication. Interestingly, NbMIP1s only shares less than 24% aa identity with MPIP1. We believe that MPIP and NbMIP1 interact with TMV MP at different infection stages. However how NbMIP1s affect virus infection remains to be revealed. Generally, J-domain proteins contain four domains. The J domain binds to Hsp70 to stimulate ATP hydrolysis [23]. The zinc finger domain specifically recognizes and binds the protein substrate, and G/F and CTD may regulate this binding [19], [54], [55]. In this study, we found that the zinc finger-CTD of NbMIP1.1a interacts with ToMV MP. According to the common transfer mechanism of substrates from J-domain proteins to Hsp70s [56], we believe that NbMIP1s may recruit and deliver TMV MP to Hsp70. Indeed, silencing of *NbHsp70c-1* also compromises TMV infection [57]. In addition, some Hsp70s facilitate proper folding of viral proteins and promote viral infectivity [58]. Further, silencing of *NbMIP1s* reduced the ToMV MP levels. NbMIP1s may be responsible for proper protein folding and maturation of TMV MP; therefore, silencing of *NbMIP1s* may result in misfolding of viral MP, which will lead to degradation of misfolded MPs. Thus NbMIP1s could affect virus infection by regulating the stability of TMV MP as Hsp70 co-chaperones during TMV infection.

### Role of NbMIP1s in Plant Immunity

Recently, a J-domain protein has been reported to be involved in plant defense and function as a positive regulator of HR-like cell death [27]. Overexpression of GmHSP40.1, a type III J domain-containing protein, caused HR-like cell death and silencing *GmHSP40.1* enhanced virus susceptibility but did not affect *R* gene mediated resistance to SMV [27]. By contrast, we found that overexpression of *NbMIP1.1a* by agroinfiltration did not cause cell death in this study (data not shown). Furthermore, silencing of *NbMIP1s* reduces TMV infection and compromises *R* gene-mediated resistance, suggesting that GmHSP40.1 and NbMIP1 regulate plant defenses by different mechanisms.

Chaperone proteins, such as Hsp90 and Hsp70, participate in *R* gene-mediated resistance and nonhost resistance [30], [41], [42], [59], [60]. Hsp90 and its cochaperones SGT1 and RAR1 regulate levels of several R proteins in plants. Silencing of *Hsp90* and *SGT1* compromises *Rx*-mediated resistance against PVX by greatly reducing the steady-state level of Rx-4HA [44], [60], [61]. SGT1 also interacts with the Prf resistance protein and is required for Prf accumulation and Prf-mediated defense signaling [47]. RAR1 controls the steady-state level of MLA in barley and RPM1 in Arabidopsis [62], [63]. In this study, we found that NbSGT1 is essential for Tm-2^2^ protein stability. However, SGT1b is also reported to antagonize RAR1 to negatively regulate accumulation of the R protein RPS5 [64]. Further, SGT1 is suggested to act as a co-chaperone for Hsp90 and Hsp70 molecular chaperones in the folding and maturation of substrate proteins including R proteins [65], [66]. In this study, we found that NbMIP1s, Tm-2^2^ and SGT1 interact with each other, and are essential for *Tm2^2^*-mediated resistance. Thus, NbMIP1s may work with SGT1 in plant immunity. J-domain proteins are generally considered to act as co-chaperones of Hsp70 [24] and SGT1 is thought to act as a co-chaperone for Hsp90 and Hsp70. Therefore, NbMIP1s and SGT1 could collaborate with cytosolic Hsp70 (and Hsp90) as co-chaperones and play a key role in the maturation of R proteins during plant resistance responses. Indeed, silencing of either *NbMIP1*s or *NbSGT1* reduces the steady-state level of Tm-2^2^ protein and causes the loss of *Tm-2^2^*-mediated resistance. In this scenario, NbMIP1s may deliver R proteins to Hsp70 in a successive manner and R proteins are the substrates of NbMIP1s and Hsp70. This is consistent with our finding that NbMIP1.1a interacts with Tm-2^2^ through its C-terminal domain including the zinc finger-CTD, a putative substrate-recognizing/binding domain and silencing of *NbMIP1s* reduces the steady-state protein level of Tm-2^2^. Silencing of *NbMIP1s* compromised the resistance mediated by several *R* genes (*N*, *Cf9* and *Pto*) and nonhost pathogen *Pst* DC3000, consistent with the observation that SGT1 is responsible for the resistance mediated by many *R* genes, as well as nonhost disease resistance [29], [46].

Besides serving as co-chaperones, J-domain proteins can act independently of its activity as the co-chaperone of Hsp70, by regulating transcriptional activation [67], [68], formation of endosomes [69], carotenoid accumulation [70], and plasma membrane H+-ATPase activity [16]. Some J-domain proteins can also be chaperones by themselves, binding to unfolded proteins and nascent peptide chains [71]. Therefore, we cannot completely rule out the possibility that NbMIP1s have additional roles in plant immunity, independent of their activity as Hsp70 co-chaperones.

As part of the Tm-2^2^ protein complex, NbMIP1s may be involved in Avr recognition by R proteins. Indeed, NbMIP1.1a interacts directly with the LRR domain of Tm-2^2^, which is involved in the recognition of a viral movement protein [28], [32]. Furthermore, NbMIP1s also interact with the corresponding Tm-2^2^ avirulence effector ToMV MP, and are required for TMV infection and plant immunity mediated by several *R* genes. Moreover, our co-IP assays, using the Tm-2^2^ EDVID mutant, suggest that Tm-2^2^, ToMV and NbMIP1s may exist in the same complex. In addition, Tm-2^2^ did not interact with ToMV MP in yeast two-hybrid assays (data not shown), suggesting that there is an indirect interaction between Tm-2^2^ and ToMV MP. These observations suggest that NbMIP1s may be a guardee of Tm-2^2^. In this scenario, NbMIP1s interact with Tm-2^2^ and are part of R protein complexes, which are targeted by ToMV MP. Under normal conditions, Tm-2^2^ associates with NbMIP1s in an inactive state. Upon ToMV infection, viral MP targets NbMIP1s, and Tm-2^2^ detects the perturbation of NbMIP1s that is induced by viral MP. This leads to Tm-2^2^ activation and the activated Tm-2^2^ activates downstream defense responses. However, we cannot rule out the possibility that NbMIP1s are not guardees of Tm-2^2^, since we used a Tm-2^2^ mutant to replace wild type Tm-2^2^ for co-IP assays and this Tm-2^2^ mutant may behave differently from wild type Tm-2^2^ in complex formation. The question of whether NbMIP1s, Tm-2^2^ and ToMV MP exist in the same complex remains a topic for future investigation.

## Materials and Methods

### Plant Materials, Pathogens and Plasmids

Wild-type *Nicotiana benthamiana*, *NN* transgenic plants, and TMV-GFP were described [29], [40]. Transgenic *Tm-2^2^ N. benthamiana* lines TM#1 and TM#5 contain the resistance gene *Tm-2^2^* with its native promoter and terminator and have extreme resistance to TMV [39] and ToMV. The pRNAi-LIC vector was described [72].

DNA fragments of ToMV MP-cLUC, TMV MP-cLUC, Tm-2^2^-cLUC, nLUC-NbMIP1s, ToMV MP-YFP, Tm-2^2^-YFP, YFP-Tm-2^2^, NbMIP1.1a-YFP, YFP-NbMIP1s, NbMIP1.1a-4×Myc, ToMV MP-3×HA, Tm-2^2^-3×HA, Tm-2^2^-4×Myc, NbSGT1-3×HA, Tm-2^2^ (VAALLA)-3×HA and ToMV MP-4×Myc were obtained by overlapping PCR. The resulting PCR products were cloned between the duplicated 35S CaMV promoter and the NOS terminator of pJG045, a pCAMBIA1300-based T-DNA vector [14]. Vectors pTRV1 [29] and pTRV2-LIC were described [29], [73]. Full-length NbMIP1.1a cDNA was PCR amplified and cloned into the pGEX4T-1 vector to express glutathione S-transferase (GST)-tagged fusion proteins in *E. coli*. ToMV MP, *Tm-2^2^* and Tm-2^2^-LRR domain were cloned into pET28a respectively to express the double-tagged ToMV MP-3×Flag-6×His, Tm-2^2^-3×Flag-6×His and Tm-2^2^ LRR-3×Flag-6×His in *E. coli*. 3′-UTR and coding sequences of *NbMIP1.1a* (JX271901: nt 1265–1431; 1–880), *NbMIP1.2* (KC791153: nt 1262–1572; 1–350), *NbMIP1.3* (KC791154: nt 1259–1439; 1–350), *NbMIP1.4b* (KC791156: nt 1259–1481; 1–350) *and NbMIP1L*(KC791157: nt 1274–1514; 1–350) were PCR amplified and cloned into pTRV2-LIC for VIGS. *NbMIP1.1a* (JX271901: nt 1–246) was PCR amplified and used to generate the hairpin RNAi construct as described [72]. All constructs were confirmed by DNA sequencing. Primer sequences are available upon request.

Constructs 35S:Cf9, 35S:Avr9, 35S:Pto, 35S:AvrPto were previously described [74]. TMV-GFP plasmid pSPDK661 was also described [40]

### Yeast Two-Hybrid Screen and Interaction Assays

The full-length ToMV *MP*, *Tm-2^2^*and *NbSGT1* genes were PCR amplified and cloned into yeast vector pYL302 to generate the LexA DNA binding domain (BD) containing bait vectors BD-ToMV MP, BD-Tm-2^2^ and BD-NbSGT1. The full-length NbMIP1s and NbMIP1.1a deletion derivates were PCR amplified and cloned into the B42 activation domain (AD)-containing vector pJG4-5. The yeast two-hybrid prey library containing tomato cDNAs was used to screen ToMV MP-binding and Tm-2^2^-LRR-binding proteins [29]. The yeast two-hybrid screen and interaction assays were performed as described [29]. For β-galactosidase assays, yeasts were incubated at 30°C with shaking (230∼250 rpm) overnight (14–18 h) in an appropriate liquid selection medium to OD_600_ = 0.5∼1. Cells of 1.0 mL of culture were pelleted by centrifugation at 14,000 rpm for 30 s. Cells were resuspended in 50 µL NET-buffer (50 mM Tris-HCl pH 8.0, 5 mM EDTA, 0.4 M NaCl, 100 U/mL Trasylol (Aprotimin), 1% Nonidet P-40 (NP-40)), 5 µL 10% Triton X-100, 1 µL 100 mM PMSF. To lyse cells, 0.2 g (ca. 100 µL) glass beads (0.5 mm in diameter) were added and the suspension was vortexed for 6 min at 4°C. 650 µL of 1× Z buffer (60 mM Na_2_HPO_4_, 40 mM NaH_2_PO_4_, 10 mM KCl, 1 mM MgSO_4_, pH = 7.0) with 0.27% (v/v) β-mercaptoethanol was added and mixed well. Next, 160 µL of ONPG (o-nitrophenyl ß D-galactopyranoside; Sigma N-1127) solution (4 mg/mL with Z-buffer) was added and also mixed well. The mixture was incubated at 30°C for 1–10 min. The reaction was quenched with 400 µL of 1 M Na_2_CO_3_. The mixture was centrifuged for 5 min at 14,000 rpm to remove cell debris. Absorbance was read at 420 nm and the relative activity was calculated using the equation: ß-galactosidase units = 1,000×(OD_420_/t×V×OD_600_), where t = time (min) of incubation of the reaction mixture, V = volume (mL) of cell culture used, OD_600_ = A_600_ of 1 mL of culture.

### GST Pull-Down Assay

GST-NbMIP1.1a, ToMV MP-3×Flag-6×His and Tm-2^2^-LRR-3×Flag-6×His fusion proteins were produced in BL21 (DE3) codon plus RIL cells. GST-NbMIP1.1a was purified using glutathione-agarose beads (Sigma) and analyzed by SDS-PAGE with Coomassie brilliant blue staining. Approximately 1 µg purified GST fusion proteins were used to pull down ToMV MP-3×Flag-6×His and Tm-2^2^-LRR-3×Flag-6×His in vitro for 3 h at room temperature. The beads were washed four times with ice-cold elution buffer (200 mM NaCl, 50 mM Tric-HCl, pH 8.0, 0.1% Triton-X100) at room temperature. The washed beads were boiled in SDS sample buffer, and proteins were separated by SDS-PAGE and detected by western blot using rabbit anti-Flag tag (Santa Cruz Biotechnology) and anti-rabbit HRP conjugated (Jackson ImmunoResearch) antibodies.

### Confocal Microscopy, Protein Extraction and Immunoblot Analysis

We used *Agrobacterium*-mediated transient expression of proteins for confocal imaging. The constructs were infiltrated into leaves of *N. benthamiana*. The leaves were detached 48 hours post infiltration (hpi), and confocal imaging was performed using an inverted Zeiss LSM 710 laser scanning microscope (Carl-Zeiss). Total, soluble and microsomal protein fractions were extracted from leaves and the immunoblot analysis as reported [38].

### Luciferase Complementation Imaging (LCI) Assays

LCI assays were performed as described [31]. All tested combinations were agroinfiltrated into leaves of *N. benthamiana*. The leaves were detached 48∼72 hpi, sprayed with 1 mM luciferin and observed under a low-light cooled CCD imaging apparatus (iXon, Andor Technology). The pictures were taken 10 minutes after exposure.

### Protein Analyses and Co-Immunoprecipitation (Co-IP)

For protein analysis, total proteins from *N. benthamiana* leaves were extracted with a ratio of 1∶2.5 of 2× Laemmli buffer [75]. Protein extracts were separated by SDS–PAGE for western blot analysis using anti-GFP (Santa Cruz Biotechnology) primary antibodies and were detected using Pierce ECL western blotting substrate (Pierce). For Co-IP assays, total proteins from *N. benthamiana* leaves (about 1 g leaf tissues for each sample) were extracted in using pre-cold IP buffer (10% (v/v) glycerol, 25 mM Tris pH 7.5, 1 mM EDTA, 150 mM NaCl), 10 mM DTT, 2% (w/v) PVPP (polyvinylpolypyrrolidone), 1× protease inhibitor cocktail, 1 mM PMSF, 0.15% (v/v) NP-40). Protein extracts were incubated with the anti-HA or anti-GFP antibodies for 2 hours at 4°C, followed by overnight incubation with protein A/G plus agarose beads (Santa Cruz Biotechnology) equilibrated with the extraction buffer. The beads were washed four times with ice-cold IP buffer at 4°C. IP samples were analyzed by SDS-PAGE, immunoblotted using anti-α-Myc, anti-HA or anti-GFP antibodies (Santa Cruz Biotechnology) and detected using Pierce ECL western blotting substrate (Pierce).

### RT-PCR and Real Time RT-PCR

Total RNA was extracted from leaves of *N. benthamiana* plants using TRNzol solution (Tiangen Biotech) and treated with RNase-free DNase I (MBI, Beijing) to remove potential DNA contamination. First-strand cDNA was synthesized using 1 µg of total RNA, oligo(dT)_15_ or gene-specific primers and M-MuLV Reverse Transcriptase according to the manufacturer's protocol (MBI). RT-PCR was performed as described [40]. Real-time RT-PCR was performed as described [76]. Primer sequences are available upon request.

### VIGS Assay, Virus Infection, and GFP Imaging

*N. benthamiana* plants were grown in pots at 25°C under a 16-h-light/8-h-dark cycle. For VIGS assays, pTRV1 and pTRV2 or its derivatives were introduced into *Agrobacterium tumefaciens* strain GV3101. VIGS assays were performed using a published protocol [40]. GFP-tagged TMV (TMV-GFP) sap was prepared from the infiltrated leaves of *N. benthamiana* plants that were infiltrated with *Agrobacterium* containing TMV-GFP plasmid pSPDK661. Each silencing experiment was repeated at least four times and each experiment included at least four independent plants. GFP was imaged under long-wavelength UV light, and photographs were taken using a Canon PowerShot A630 digital camera.

### Virus Replication Assay

Virus were isolated at 6 days post infection (dpi) from *N. benthamina* leaves inoculated with TMV-GFP and purified with PEG according to a published protocol [77]. For extraction of TMV-GFP RNA, the purified virus samples were treated with phenol and sodium dodecyl sulfate and followed by ethanol precipitation [77], [78]. *N. benthamiana* mesophyll protoplasts from VIGS plants were prepared as described [79] with slight modification [80]. In every infection assay, a 1 mL aliquot of up to 10^5^ protoplasts was transfected with 2 µg TMV-GFP RNA by the PEG-mediated method. The transfected protoplasts were cultured at 28°C as 1 mL suspensions. For detection of TMV replication, at 0, 24, 48 and 72 hours post transfection total RNA was extracted with TRNzol solution (Tiangen Biotech) from inoculated protoplast cultures and reverse transcribed with TMV specific primers. Real time RT-PCR analyses were then conducted, using *Actin* as an internal control.

### Pathogen-Induced Cell Death Assay

Pathogen-induced cell death assays were as described [74]. *N. benthamiana* plants were were infiltrated with Agrobacterium carrying 35S:Cf9 and 35S:Avr9 or 35S:Pto and 35S:AvrPto. Agrobacterium cultures were mixed in a 1∶1 ratio while maintaining an OD_600_ = 0.5. Pst DC3000 were resuspended in 10 mM MgCl_2_ to OD_600_ = 0.4 and infiltrated into leaves. For Tm-2^2^ induced HR assays, Tm-2^2^-HA or Tm-2^2^ (VAALLA)-HA were agroinfiltrated into wildtype *N. benthamiana* leaves with Agrobacterium carrying 35S:ToMV MP-Myc.

### *Pst* DC3000 Growth

For measurement of *Pst* DC3000 growth, pathogen was resuspended in 10 mM MgCl_2_ (10,000-fold dilution from OD_600_ = 1) and infiltrated into leaves. Four discs of 6 mm diameter were collected per sample at the indicated time points and used to count the pathogen titer as described [46].

### Accession Numbers

Sequence data from this article can be found in the GenBank database under the following accession numbers: NbMIP1.1a (JX271901), NbMIP1.1b (KC791152), NbMIP1.2 (KC791153), NbMIP1.3 (KC791154), NbMIP1.4a (KC791155), NbMIP1.4b (KC791156), NbMIP1L1 (KC791157), Tm-2^2^ (AF536201.1), ToMV MP (X02144), NbSGT1 (AF494083.1).

## Supporting Information

Figure S1 **Alignment of the NbMIP1.1a amino acid sequence with its homologs.** Homologs from *N. benthamiana* NbMIP1.1b (KC791152), NbMIP1.2 (KC791153), NbMIP1.3 (KC791154), NbMIP1.4a (KC791155), NbMIP1.4b (KC791156), NbMIP1L1 (KC791157), tobacco (NtJ; DFCI: TC123211), tomato (SlMIP1; DFCI: TC192697), Arabidopsis (AtJ3; At3g44110), human (HsDJ2; NP_005871.1), yeast (YDJ1; X56560.1) and *E. coli* (EcdnaJp; X56560.1) are included. The alignment was generated using Clustal W2. Black, dark gray, light gray and white backgrounds represent residues that are conserved in 100%, above 80%, above 60%, or below 60% of the sequences at the corresponding positions. Capital letters under each block indicate residues that are consensus in all aligned sequences and the lowercase letters indicate mostly conserved residues other than consensus ones. The black lines above the sequence alignment indicate the position of conserved domains. Numbers at the right indicate the positions of amino acid residues.
(TIF)

Figure S2 **The inputs of purified GST-fusion proteins and Flag-tagged proteins in pull-down assays.** (**A**) The glutathione beads immobilized with GST-NbMIP1.1a or GST were separated by SDS-PAGE and stained with Coomassie brilliant blue. Black arrows indicate the corresponding bands of GST-NbMIP1.1a and GST respectively. (**B**) The ToMV MP-3×Flag-6×His and Tm-2^2^-LRR-3×Flag-6×His were separated by SDS-PAGE and stained with Coomassie brilliant blue. Black arrows indicate the corresponding bands of ToMV MP-3×Flag-6×His and Tm-2^2^-LRR-3×Flag-6×His respectively.
(TIF)

Figure S3 **The C-terminal zinc finger and CTD domains of NbMIP1.1a are responsible for the interactions with ToMV MP and Tm-2^2^.** (**A**) Diagram of the NbMIP1.1a truncations used in the yeast two-hybrid analysis as B42 activation domain (AD) fusion vectors. (**B**) Yeast two-hybrid analysis of the interactions of NbMIP1.1a truncated derivatives with ToMV MP (upper row) or Tm-2^2^ (lower row). The zinc finger-CTD domains of NbMIP1.1a are required for its binding to ToMV MP and Tm-2^2^. Yeast transformed with AD-NbMIP1.1a/BD, AD/BD-ToMV MP, AD/BD-Tm-2^2^ served as negative controls. (**C**) Quantification of β-galactosidase activity in yeast two-hybrid interactions.
(TIF)

Figure S4 **LIC assay to show that NbMIP1.1a interacts with TMV MP** ***in vivo*** **.** Image shown is luminescence of a *N. benthamiana* leaf that was agro-infiltrated with nLUC-NbMIP1.1a and TMV MP-cLUC. The combinations of nLUC-NbMIP1.1a and cLUC, nLUC and TMV MP-cLUC were included as negative controls.
(TIF)

Figure S5 **NbMIP1s interact with ToMV MP and Tm-2^2^ in yeast and in plants.** (**A–D**) Yeast two-hybrid analysis of the interactions of NbMIP1s with ToMV MP (A, B) or Tm-2^2^ (C, D). (A, C) Growth of yeast strains on Leu^−^ selection medium containing Gal/Raf. Serial dilutions of yeast cultures at 1, 10^−1^, 10^−2^ were spotted onto Leu^−^ plates and grew for 5 days at 28°C. (B, D) Quantification of the β-galactosidase activity in yeast two-hybrid interactions. (**E, F**) LIC assay to show that NbMIP1s interact with ToMV MP (E) or Tm-2^2^ (F) *in vivo*. Images shown are luminescence of *N. benthamiana* leaves that were agro-infiltrated with nLUC-NbMIP1s and ToMV MP-cLUC or Tm-2^2^-cLUC. The combinations of nLUC/TMV MP-cLUC, nLUC/Tm-2^2^-cLUC were included as the negative controls.
(TIF)

Figure S6 **The subcellular localization of NbMIP1.1a-YFP in** ***N. benthamiana*** **cells.** (**A**) NbMIP1.1a-YFP was transiently expressed in leaves of *N. benthamiana* via agroinfiltration and the image was taken at 48 hpi. PCD3-1002 is a CFP tagged plasma membrane marker. DAPI: staining for nuclei. Scale bar represents 20 µm. (**B**) NbMIP1.1a-YFP was found in both the soluble fraction and the membrane fraction (upper panel). Protein extracts were centrifuged at 100,000×g to produce crude soluble (S100) and microsomal (P100) fractions. Fractions were analyzed by western blotting following separation by SDS-PAGE. The gels were probed using anti-GFP, anti-V-H-ATPase (vacuolar H-ATPase subunit, a vacuolar membrane marker) and anti-PEPC (phosphoenolpyruvate carboxylase, a cytosolic marker) antibodies, as indicated.
(TIF)

Figure S7 **The subcellular localization of other YFP-tagged NbMIP1s in** ***N. benthamiana*** **cells.** (**A**) YFP-tagged NbMIP1s were transiently expressed in leaves of *N. benthamiana* via Agrobacteria infiltration and images were taken at 48 hpi. DAPI: staining for nuclei. Scale bar represents 20 µm. (**B**) YFP-NbMIP1s were found in both soluble (S100) and microsomal (P100) fractions (upper panel). The bands using anti-GFP, anti-V-H-ATPase and anti-PEPC antibodies were as indicated.
(TIF)

Figure S8 **The subcellular localization of NbMIP1.1a did not change during** ***Tm-2^2^*** **-mediated resistance.** (**A**) YFP-NbMIP1.1a was transiently coexpressed with ToMV or TMV respectively in leaves of TM#1 via agroinfiltration and images were taken at 72 hpi. DAPI: staining for nuclei. Scale bar represents 20 µm. (**B**) YFP-NbMIP1.1a was still found in both soluble (S100) and microsomal (P100) fractions (upper panel) upon ToMV or TMV infection. The bands using anti-GFP, anti-V-H-ATPase and anti-PEPC antibodies are as indicated.
(TIF)

Figure S9 ***NbMIP1s*** **were induced upon TMV infection.** Real-time RT-PCR showed that the *NbMIP1s* mRNA levels increased in TMV-GFP infected plants; *Actin* mRNA levels were used as internal controls. Data are shown as means ± SD for 3 independent triplicate experiments (**P<0.01, Student's *t*-test).
(TIF)

Figure S10 ***Tm-2^2^*** **transgenic** ***N. benthamiana*** **lines TM#1 and TM#5 confer extreme resistance against ToMV.** The shoots of the wild type *N. benthamiana* plant, but not TM#1 and TM#5 plants, became curled 7 days post ToMV infection (dpi). Scale bars represent 1 cm.
(TIF)

Figure S11 **TMV MP specifically induced the HR in** ***Tm-2^2^*** **transgenic** ***N. benthamiana*** **line TM#1.** TMV MP, but not helicase domain (P50) of TMV replicase, can induce an HR when expressed in the *Tm-2^2^* transgenic *N. benthamiana* line.
(TIF)

Figure S12 ***NbMIP1s*** **were induced during** ***Tm-2^2^*** **-mediated TMV resistance in** ***N. benthamiana*** **.** Real-time RT-PCR showing the *NbMIP1s* mRNA level increased after TMV-GFP infection in TM#1 plants. *Actin* was used as the internal control. Data are shown as means ± SD for 3 independent triplicate experiments (**P<0.01, Student's *t*-test).
(TIF)

Figure S13 **Silencing of individual** ***NbMIP1s*** **had no effect on** ***Tm-2^2^*** **-mediated resistance against TMV infection.** (**A**) Real time RT-PCR to confirm the specific suppression of *NbMIP1s*, with *Actin* mRNA levels as an internal control. Data are shown as means ± SD for 3 independent triplicate experiments (**P<0.01, Student's *t*-test). (**B**) Silencing of individual *NbMIP1s* using their gene-specific 3′-UTRs had no effect on *Tm-2^2^*-mediated resistance against TMV infection. Neither TMV-GFP infection foci nor visible HR lesions were observed in the inoculated leaves of individual *NbMIP1s* silenced TM#1 plants. Scale bars represent 1 cm.
(TIF)

Figure S14 ***NbMIP1s*** **are required for** ***Tm-2^2^*** **-mediated resistance against ToMV.** (**A**) Silencing of *NbMIP1s* caused the appearance of visible necrotic lesions in inoculated leaves of *Tm-2^2^* transgenic *N. benthamiana* TM#1 plants 4 days post ToMV infection (dpi). (**B**) ToMV induced systemic necrosis in *NbMIP1s*-silenced but not in non-silenced TRV control TM#1 plants 14 dpi. Scale bars represent 1 cm. (**C**) RT-PCR to confirm the presence of ToMV in systemic leaves of *NbMIP1s*-silenced TM#1 plants.
(TIF)

Figure S15 ***NbMIP1s*** **are required for** ***Tm-2^2^*** **-mediated resistance against TMV-GFP.** (**A**) Real time RT-PCR to confirm the suppression of *NbMIP1s* using the coding sequences of *NbMIP1s*; *Actin* mRNA levels were used as the internal control. (**B**) Silencing of *NbMIP1s* using the coding sequence of *NbMIP1.2* (*NbMIP1.2* VIGS) or *NbMIP1.3* (*NbMIP1.3* VIGS) caused downward-curled leaves. (**C**) Silencing of *NbMIP1s* using the coding sequence of *NbMIP1.2*, *NbMIP1.3* and *NbMIP1.4b* (*NbMIP1.4b* VIGS) caused the appearance of TMV-GFP infection foci and visible HR lesions in the inoculated leaves of *NbMIP1s*-silenced *Tm-2^2^*-containing TM#1 plants. Scale bars represent 1 cm. (**D**) Silencing of *NbMIP1s* using the coding sequence of *NbMIP1.2* and *NbMIP1.3* compromised *Tm-2^2^*-mediated resistance against TMV, and TMV-GFP spread from the inoculated leaves into the upper non-inoculated leaves. TRV-infected TM#1 plants were used as negative controls. Photos were taken at 16 dpi. Scale bars represent 1 cm. (**E–F**) RT-PCR was performed to confirm the presence of TMV-GFP in local leaves (E) and systemic leaves (F) in *NbMIP1.2* VIGS and *NbMIP1.3* VIGS TM#1 plants.
(TIF)

Figure S16 **Silencing of** ***NbMIP1s*** **with** ***NbMIP1*** **hairpin RNAi construct by agroinfiltration compromised** ***Tm-2^2^*** **-mediated resistance against TMV.** (**A**) Real-time RT-PCR confirmed the suppression of *NbMIP1s* in *NbMIP1* hairpin RNAi TM#1 plants, and *Actin* mRNA levels were used as the internal control. Data are shown as means ± SD for 3 independent triplicate experiments (**P<0.01, Student's *t*-test). (**B**) Compared to the vector control plants, *NbMIP1* hairpin RNAi TM#1 plants have no visible developmental phenotype. (**C**) Silencing of *NbMIP1s* using *NbMIP1* hairpin RNAi compromised *Tm-2^2^*-mediated resistance against TMV, and caused TMV-GFP spreading into the upper non-inoculated leaves of TM#1 plants. The empty RNAi vector infected TM#1 plants was used as the negative control. Photos were taken at 18 dpi. Scale bars represent 1 cm. (**D**) RT-PCR was performed to confirm the presence of TMV-GFP in systemic leaves of *NbMIP1* hairpin RNAi TM#1 plants.
(TIF)

Figure S17 **Silencing of individual** ***NbMIP1s*** **did not affect TMV infection.** (**A–B**) Silencing of individual *NbMIP1s* using their gene-specific 3′-UTR had no effect on cell-to-cell movement (A) and systemic movement (B) of TMV. Photos were taken at 3 dpi and 5 dpi respectively. Scale bars represent 1 cm.
(TIF)

Figure S18 **Silencing** ***NbMIP1s*** **had no effect on TMV replication.** Real-time RT-PCR showed that TMV-GFP effectively replicated in protoplasts derived from *N. benthamiana* plants during TMV infection, but suppression of *NbMIP1s* (*NbMIP1* VIGS) had no significant effect on either TMV-GFP genomic vRNA level (A) or TMV-GFP CP vRNA level (B). *Actin* mRNA level was used as the internal control. Data are shown as means ± SD for 3 independent triplicate experiments (Student's *t*-test).
(TIF)

Figure S19 **Tm-2^2^-YFP and ToMV MP-YFP can induce the HR.** (**A**) C-terminal YFP tagged Tm-2^2^ (Tm-2^2^-YFP) induced HR when co-expressed with ToMV MP. Tm-2^2^ plus ToMV MP, Tm-2^2^ and ToMV MP were used as positive and negative controls, respectively. (**B**) C-terminal YFP tagged ToMV MP (ToMV MP-YFP) induced HR when co-expressed with Tm-2^2^. Tm-2^2^ plus ToMV MP and Tm-2^2^ served as positive and negative controls, respectively.
(TIF)

Figure S20 **NbMIP1s are essential for the stability of ToMV MP in transgenic** ***ToMV MP-Myc*** **plants.** (**A**) Silencing of *NbMIP1s* greatly reduced ToMV MP protein levels in transgenic plants constitutively expressing ToMV MP-Myc. Proteins were extracted from the leaf tissues of *NbMIP1s*-silenced and TRV-only infected transgenic ToMV *MP-Myc* plants respectively at 14 days post agroinfiltration for VIGS, and then followed by western blot analysis with anti-Myc antibody. Ponceau Red staining of RuBisCO indicates equal loading (lower panel). All experiments were performed three times with three replicated samples in each experiment. (**B**) The real-time RT-PCR showed that silencing of *NbMIP1s* had no effect on the mRNA level of *MP-Myc*, *MP-YFP* and *Tm-2^2^-YFP*, and *Actin* mRNA levels were used as internal controls. Data are shown as means ± SD for 3 independent triplicate experiments (Student's *t*-test).
(TIF)

Figure S21 **LCI assays show that NbMIP1s interact with NbSGT1 in plants.** Images shown are luminescence of *N. benthamiana* leaves that were agro-infiltrated with NbSGT1-nLUC and NbMIP1s-cLUC. The combination of NbSGT1-nLUC and cLUC was included as the negative control.
(TIF)
