# Supplementary figures and images for: Type I J-Domain NbMIP1 Proteins Are Required for Both Tobacco Mosaic Virus Infection and Plant Innate Immunity
Source: PLoS Pathog. 2013 Oct 3;9(10):e1003659. doi: 10.1371/journal.ppat.1003659 (PMC3789785; doi:10.1371/journal.ppat.1003659)

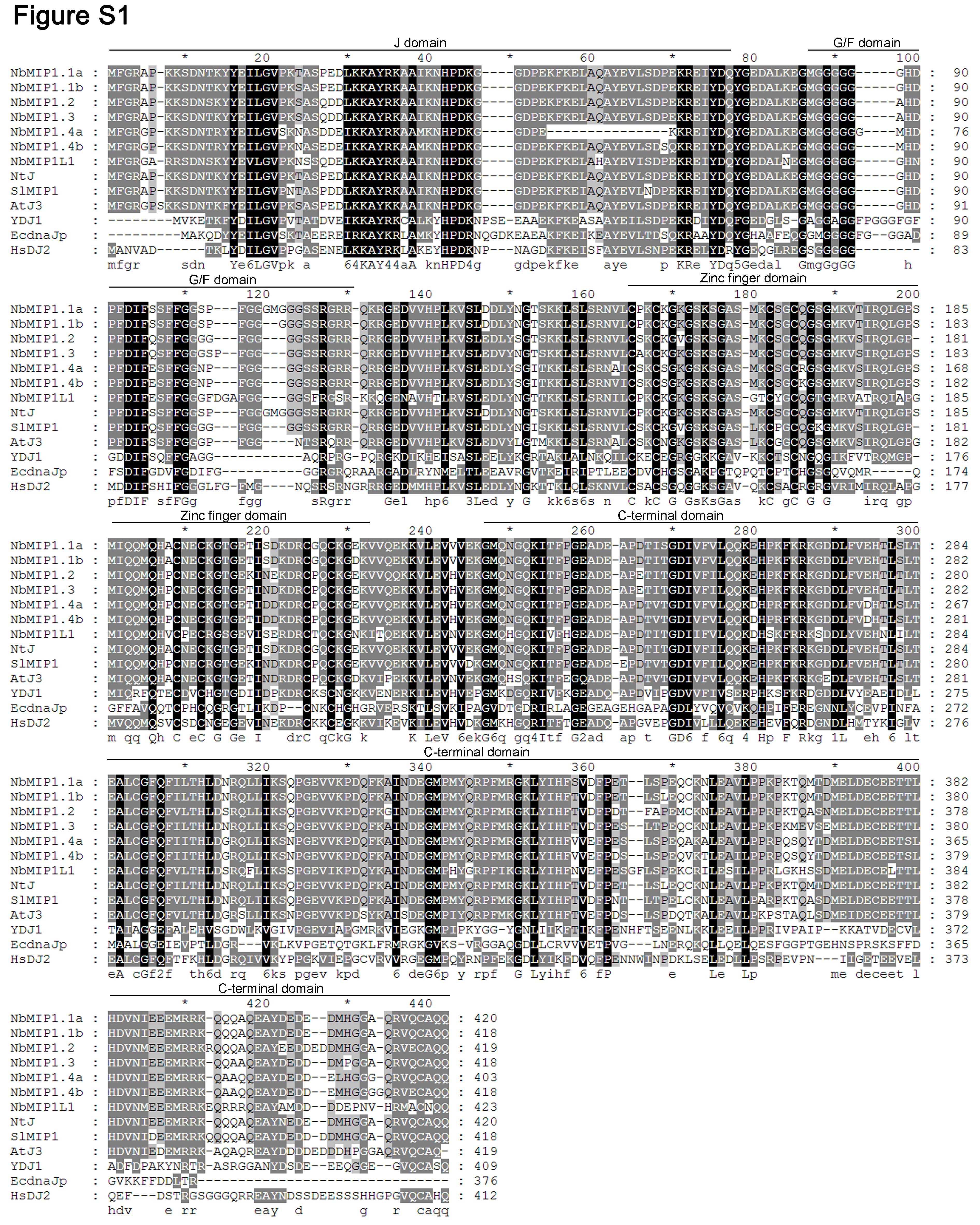

Supplement: Figure S1 — Alignment of the NbMIP1.1a amino acid sequence with its homologs. Homologs from N. benthamiana NbMIP1.1b (KC791152), NbMIP1.2 (KC791153), NbMIP1.3 (KC791154), NbMIP1.4a (KC791155), NbMIP1.4b (KC791156), NbMIP1L1 (KC791157), tobacco (NtJ; DFCI: TC123211), tomato (SlMIP1; DFCI: TC192697), Arabidopsis (AtJ3; At3g44110), human (HsDJ2; NP_005871.1), yeast (YDJ1; X56560.1) and E. coli (EcdnaJp; X56560.1) are included. The alignment was generated using Clustal W2. Black, dark gray, light gray and white backgrounds represent residues that are conserved in 100%, above 80%, above 60%, or below 60% of the sequences at the corresponding positions. Capital letters under each block indicate residues that are consensus in all aligned sequences and the lowercase letters indicate mostly conserved residues other than consensus ones. The black lines above the sequence alignment indicate the position of conserved domains. Numbers at the right indicate the positions of amino acid residues. (TIF) [file ppat.1003659.s001.tif]

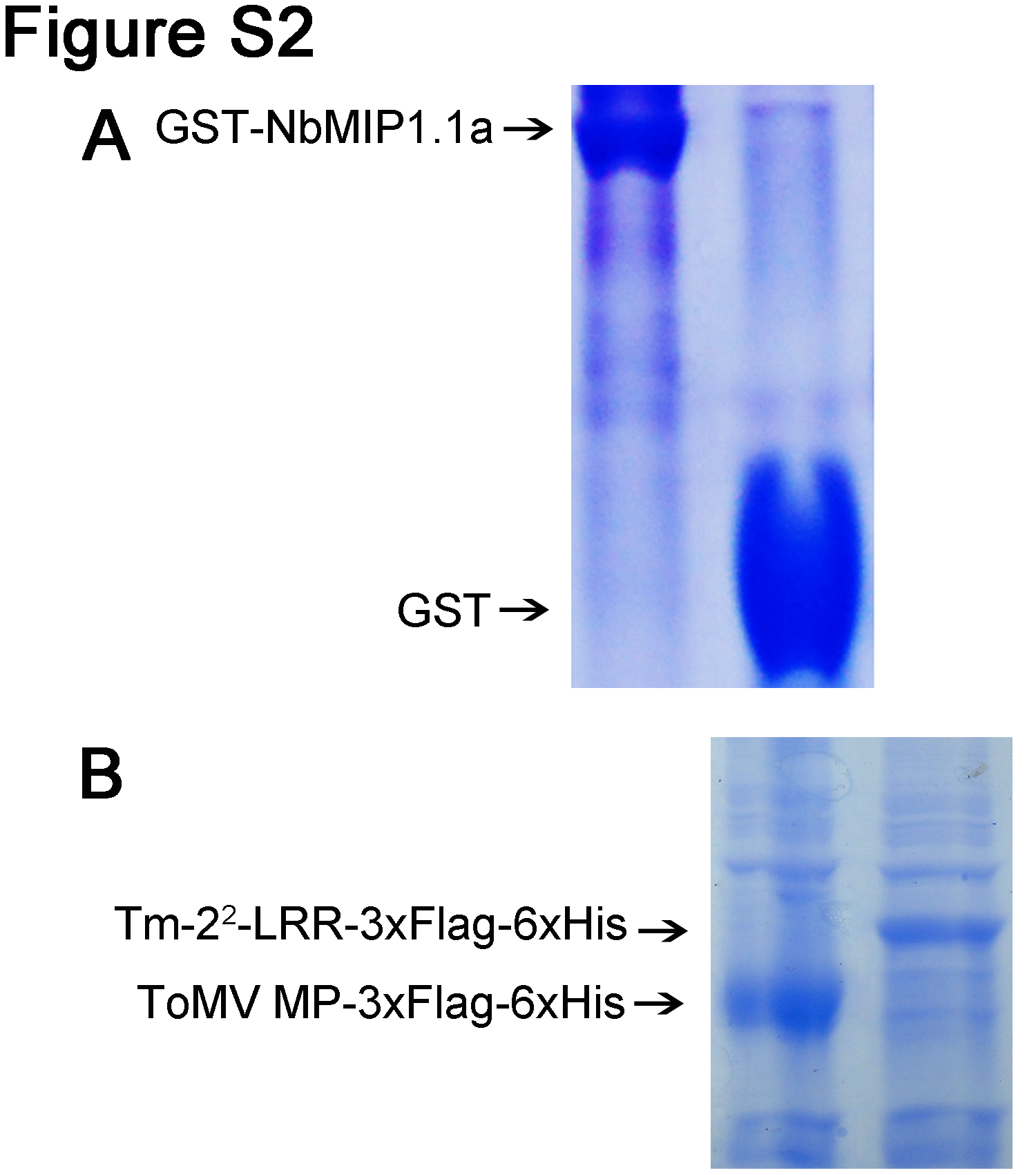

Supplement: Figure S2 — The inputs of purified GST-fusion proteins and Flag-tagged proteins in pull-down assays. (A) The glutathione beads immobilized with GST-NbMIP1.1a or GST were separated by SDS-PAGE and stained with Coomassie brilliant blue. Black arrows indicate the corresponding bands of GST-NbMIP1.1a and GST respectively. (B) The ToMV MP-3×Flag-6×His and Tm-22-LRR-3×Flag-6×His were separated by SDS-PAGE and stained with Coomassie brilliant blue. Black arrows indicate the corresponding bands of ToMV MP-3×Flag-6×His and Tm-22-LRR-3×Flag-6×His respectively. (TIF) [file ppat.1003659.s002.tif]

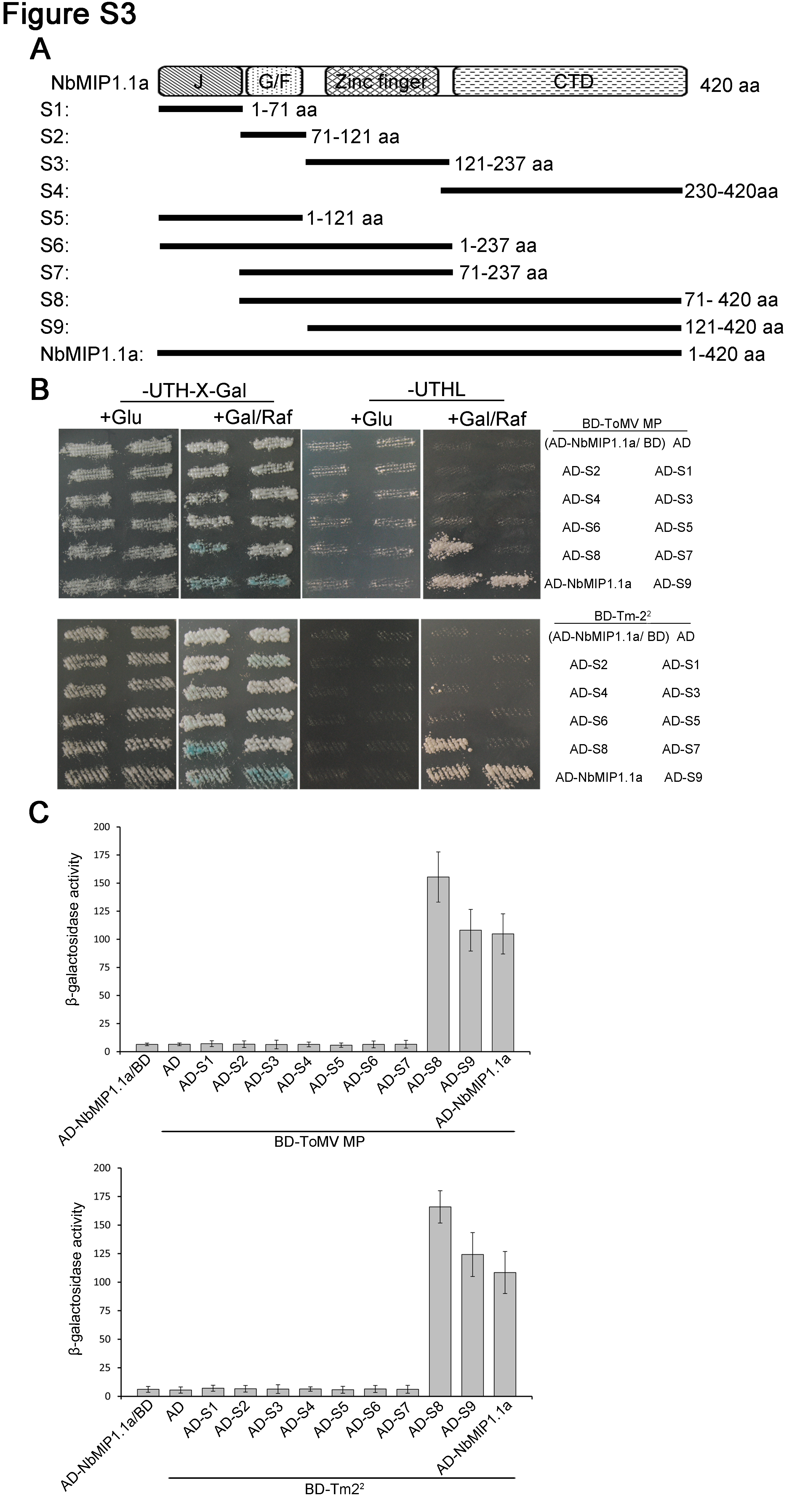

Supplement: Figure S3 — The C-terminal zinc finger and CTD domains of NbMIP1.1a are responsible for the interactions with ToMV MP and Tm-22. (A) Diagram of the NbMIP1.1a truncations used in the yeast two-hybrid analysis as B42 activation domain (AD) fusion vectors. (B) Yeast two-hybrid analysis of the interactions of NbMIP1.1a truncated derivatives with ToMV MP (upper row) or Tm-22 (lower row). The zinc finger-CTD domains of NbMIP1.1a are required for its binding to ToMV MP and Tm-22. Yeast transformed with AD-NbMIP1.1a/BD, AD/BD-ToMV MP, AD/BD-Tm-22 served as negative controls. (C) Quantification of β-galactosidase activity in yeast two-hybrid interactions. (TIF) [file ppat.1003659.s003.tif]

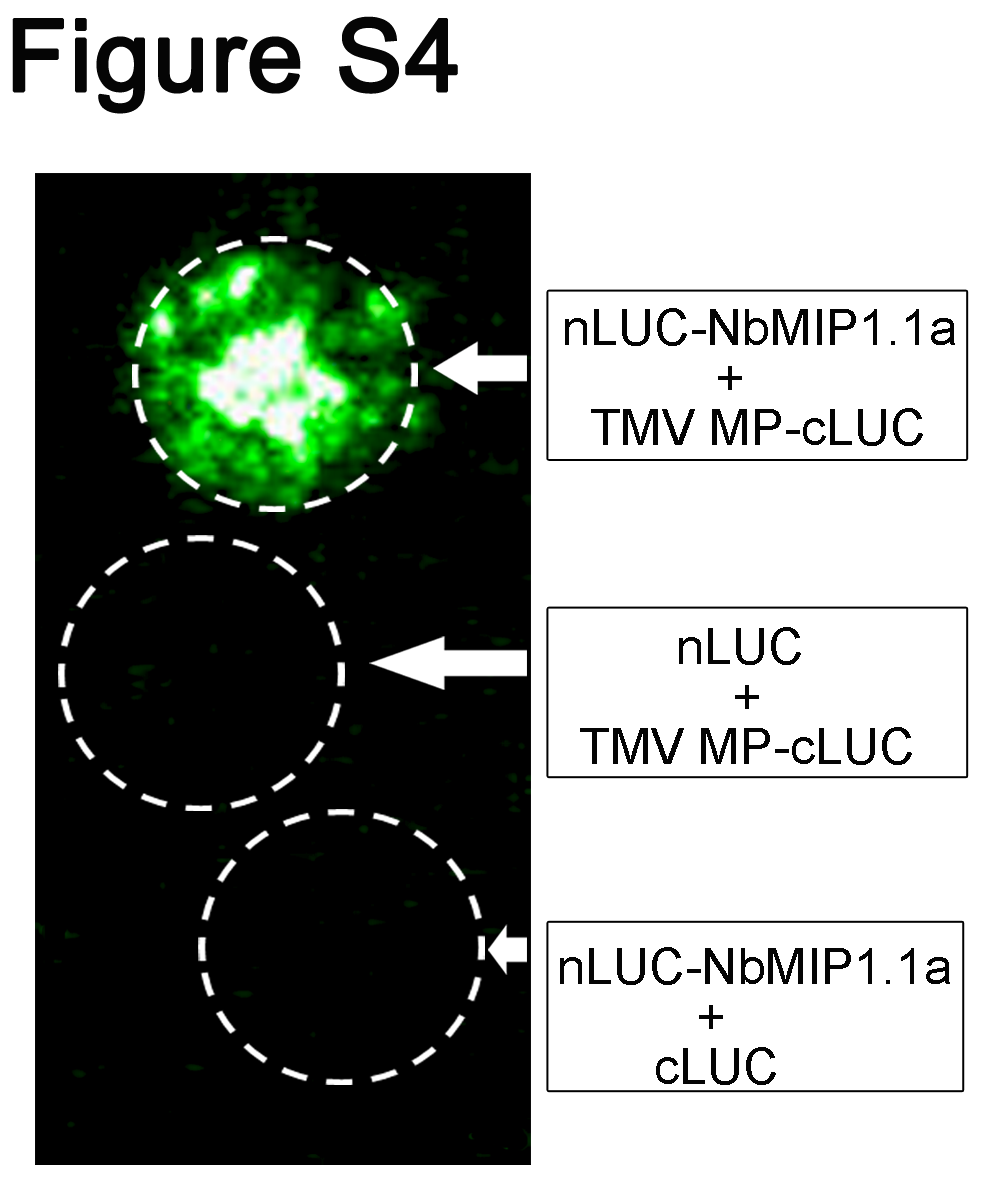

Supplement: Figure S4 — LIC assay to show that NbMIP1.1a interacts with TMV MP in vivo . Image shown is luminescence of a N. benthamiana leaf that was agro-infiltrated with nLUC-NbMIP1.1a and TMV MP-cLUC. The combinations of nLUC-NbMIP1.1a and cLUC, nLUC and TMV MP-cLUC were included as negative controls. (TIF) [file ppat.1003659.s004.tif]

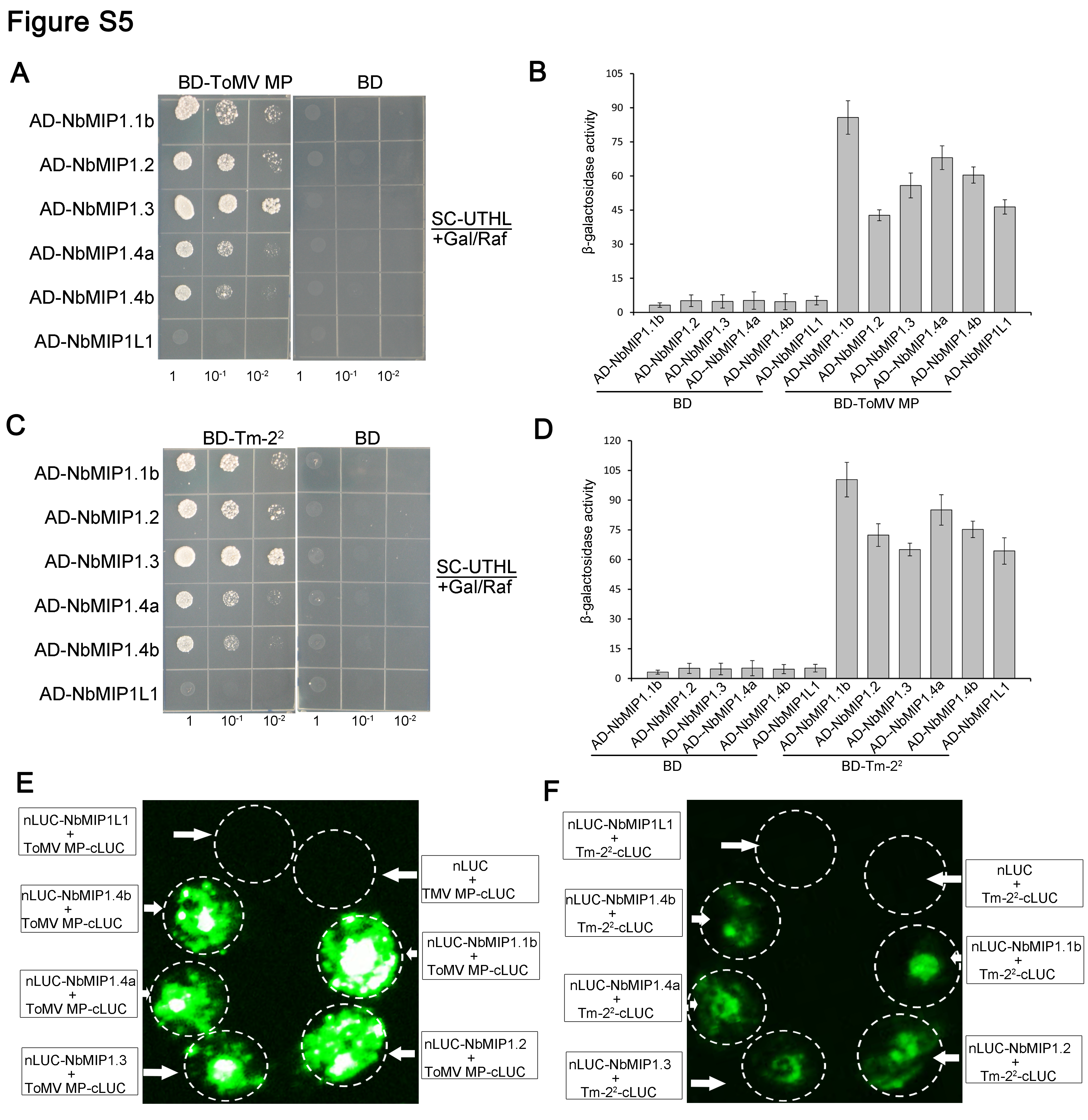

Supplement: Figure S5 — NbMIP1s interact with ToMV MP and Tm-22 in yeast and in plants. (A–D) Yeast two-hybrid analysis of the interactions of NbMIP1s with ToMV MP (A, B) or Tm-22 (C, D). (A, C) Growth of yeast strains on Leu− selection medium containing Gal/Raf. Serial dilutions of yeast cultures at 1, 10−1, 10−2 were spotted onto Leu− plates and grew for 5 days at 28°C. (B, D) Quantification of the β-galactosidase activity in yeast two-hybrid interactions. (E, F) LIC assay to show that NbMIP1s interact with ToMV MP (E) or Tm-22 (F) in vivo. Images shown are luminescence of N. benthamiana leaves that were agro-infiltrated with nLUC-NbMIP1s and ToMV MP-cLUC or Tm-22-cLUC. The combinations of nLUC/TMV MP-cLUC, nLUC/Tm-22-cLUC were included as the negative controls. (TIF) [file ppat.1003659.s005.tif]

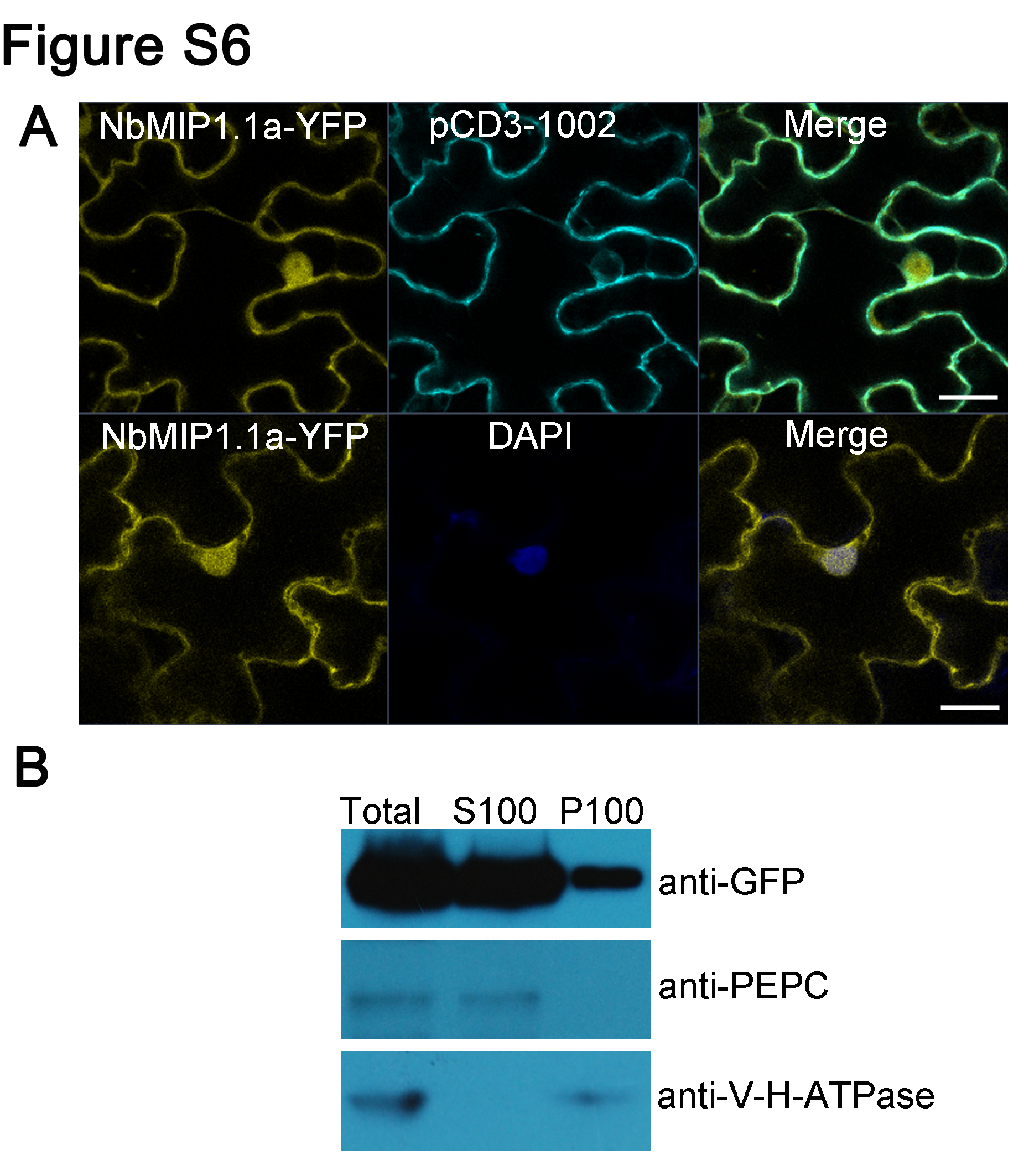

Supplement: Figure S6 — The subcellular localization of NbMIP1.1a-YFP in N. benthamiana cells. (A) NbMIP1.1a-YFP was transiently expressed in leaves of N. benthamiana via agroinfiltration and the image was taken at 48 hpi. PCD3-1002 is a CFP tagged plasma membrane marker. DAPI: staining for nuclei. Scale bar represents 20 µm. (B) NbMIP1.1a-YFP was found in both the soluble fraction and the membrane fraction (upper panel). Protein extracts were centrifuged at 100,000×g to produce crude soluble (S100) and microsomal (P100) fractions. Fractions were analyzed by western blotting following separation by SDS-PAGE. The gels were probed using anti-GFP, anti-V-H-ATPase (vacuolar H-ATPase subunit, a vacuolar membrane marker) and anti-PEPC (phosphoenolpyruvate carboxylase, a cytosolic marker) antibodies, as indicated. (TIF) [file ppat.1003659.s006.tif]

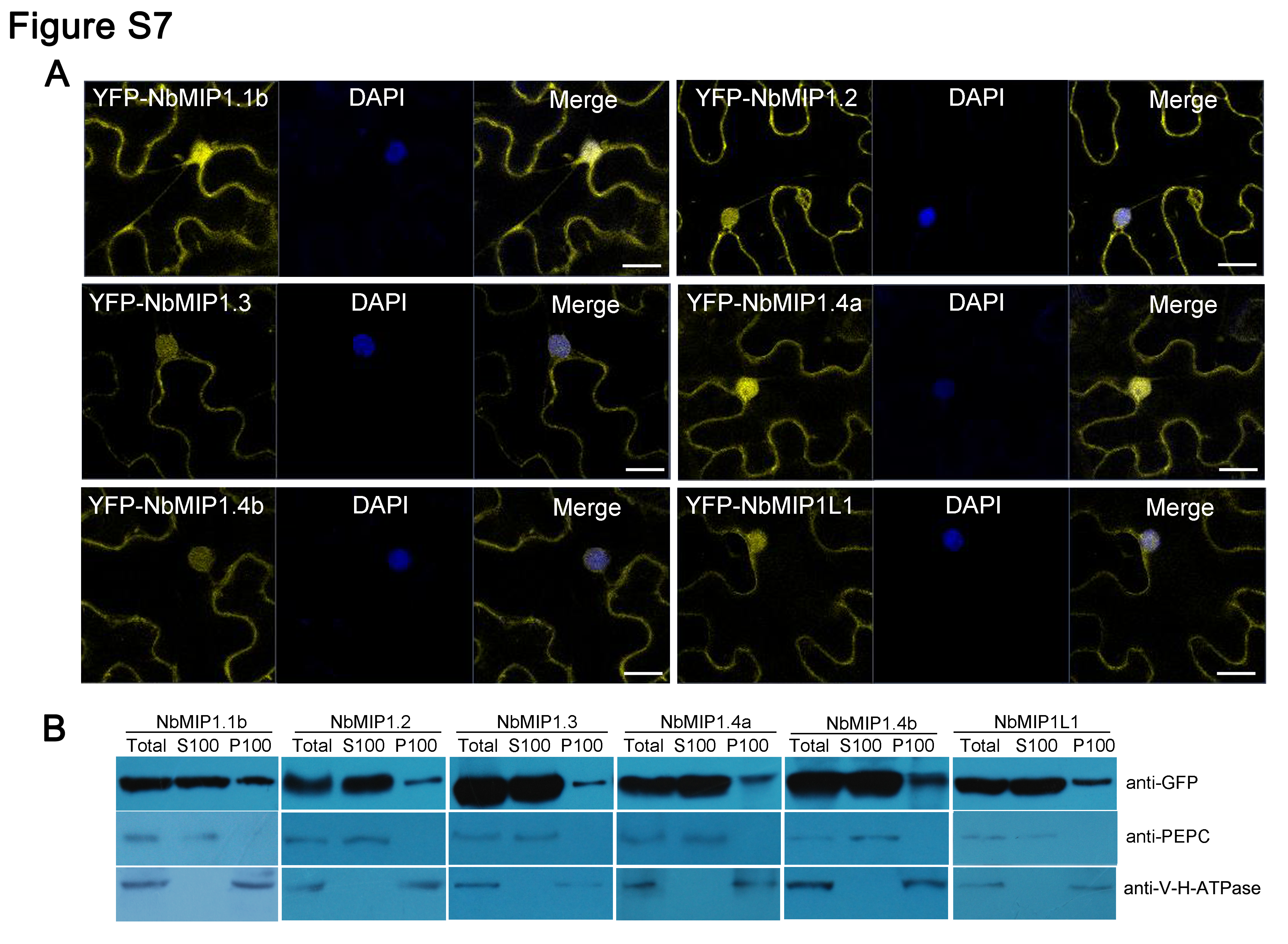

Supplement: Figure S7 — The subcellular localization of other YFP-tagged NbMIP1s in N. benthamiana cells. (A) YFP-tagged NbMIP1s were transiently expressed in leaves of N. benthamiana via Agrobacteria infiltration and images were taken at 48 hpi. DAPI: staining for nuclei. Scale bar represents 20 µm. (B) YFP-NbMIP1s were found in both soluble (S100) and microsomal (P100) fractions (upper panel). The bands using anti-GFP, anti-V-H-ATPase and anti-PEPC antibodies were as indicated. (TIF) [file ppat.1003659.s007.tif]

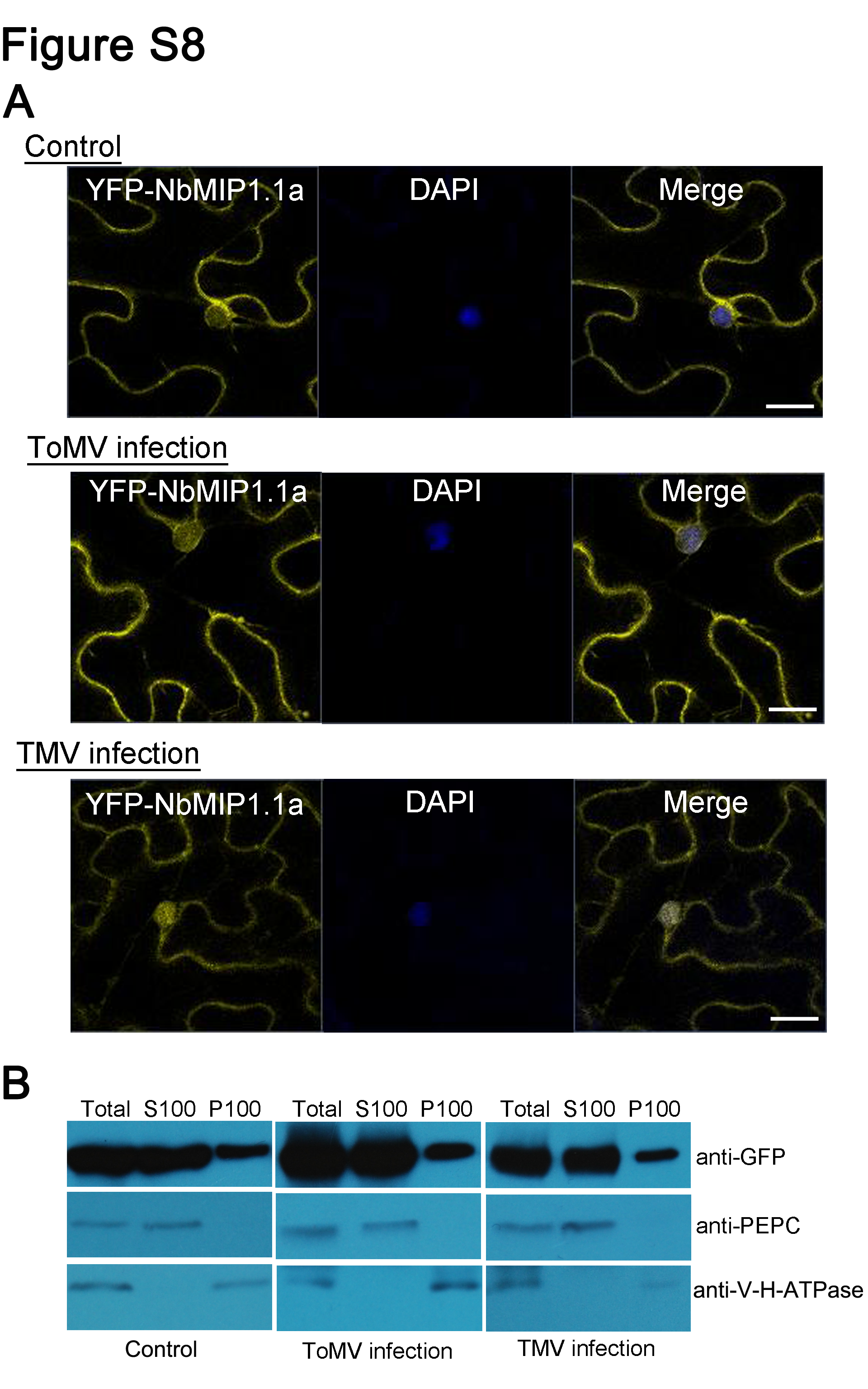

Supplement: Figure S8 — The subcellular localization of NbMIP1.1a did not change during Tm-22 -mediated resistance. (A) YFP-NbMIP1.1a was transiently coexpressed with ToMV or TMV respectively in leaves of TM#1 via agroinfiltration and images were taken at 72 hpi. DAPI: staining for nuclei. Scale bar represents 20 µm. (B) YFP-NbMIP1.1a was still found in both soluble (S100) and microsomal (P100) fractions (upper panel) upon ToMV or TMV infection. The bands using anti-GFP, anti-V-H-ATPase and anti-PEPC antibodies are as indicated. (TIF) [file ppat.1003659.s008.tif]

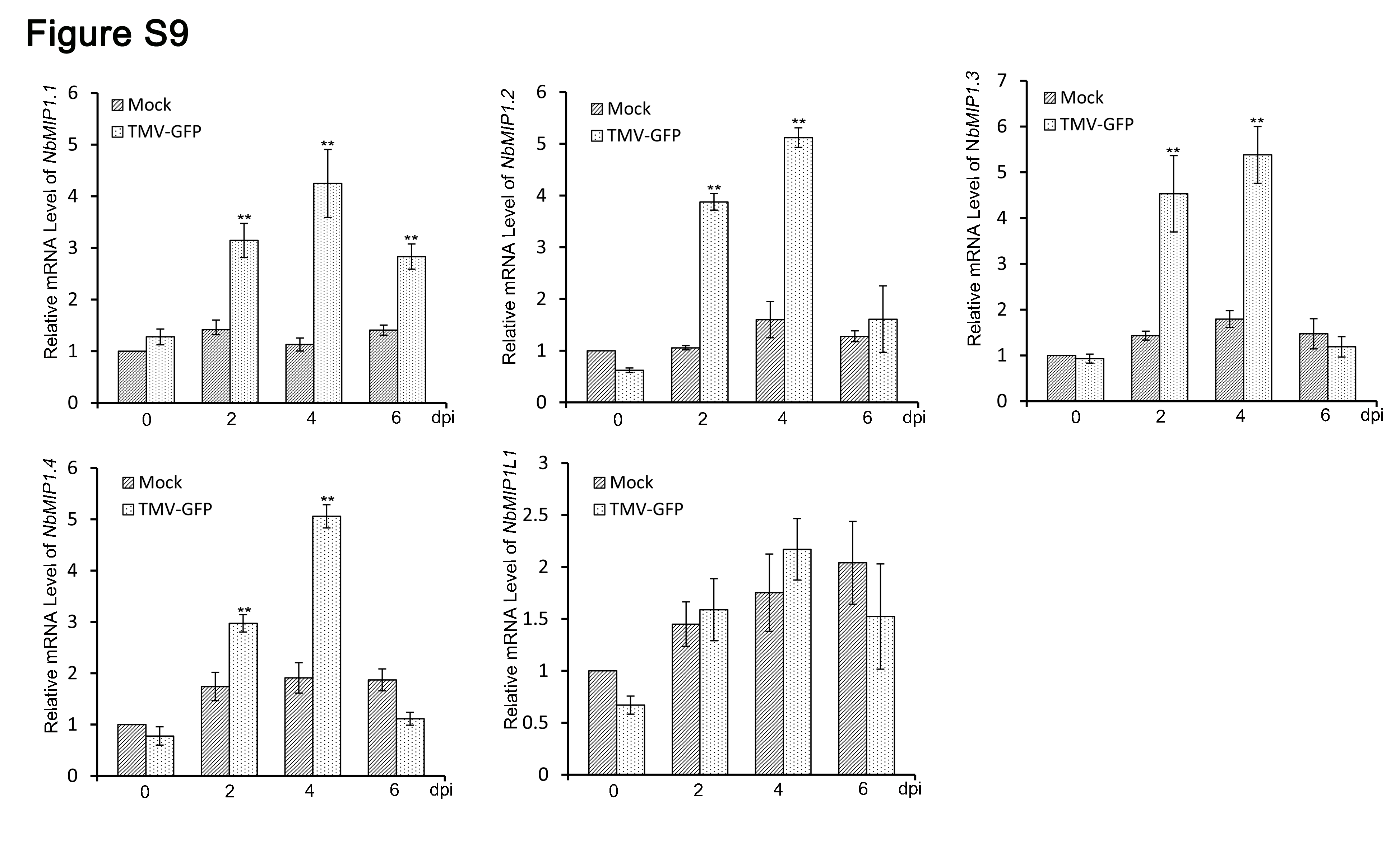

Supplement: Figure S9 — NbMIP1s were induced upon TMV infection. Real-time RT-PCR showed that the NbMIP1s mRNA levels increased in TMV-GFP infected plants; Actin mRNA levels were used as internal controls. Data are shown as means ± SD for 3 independent triplicate experiments (**P<0.01, Student's t-test). (TIF) [file ppat.1003659.s009.tif]

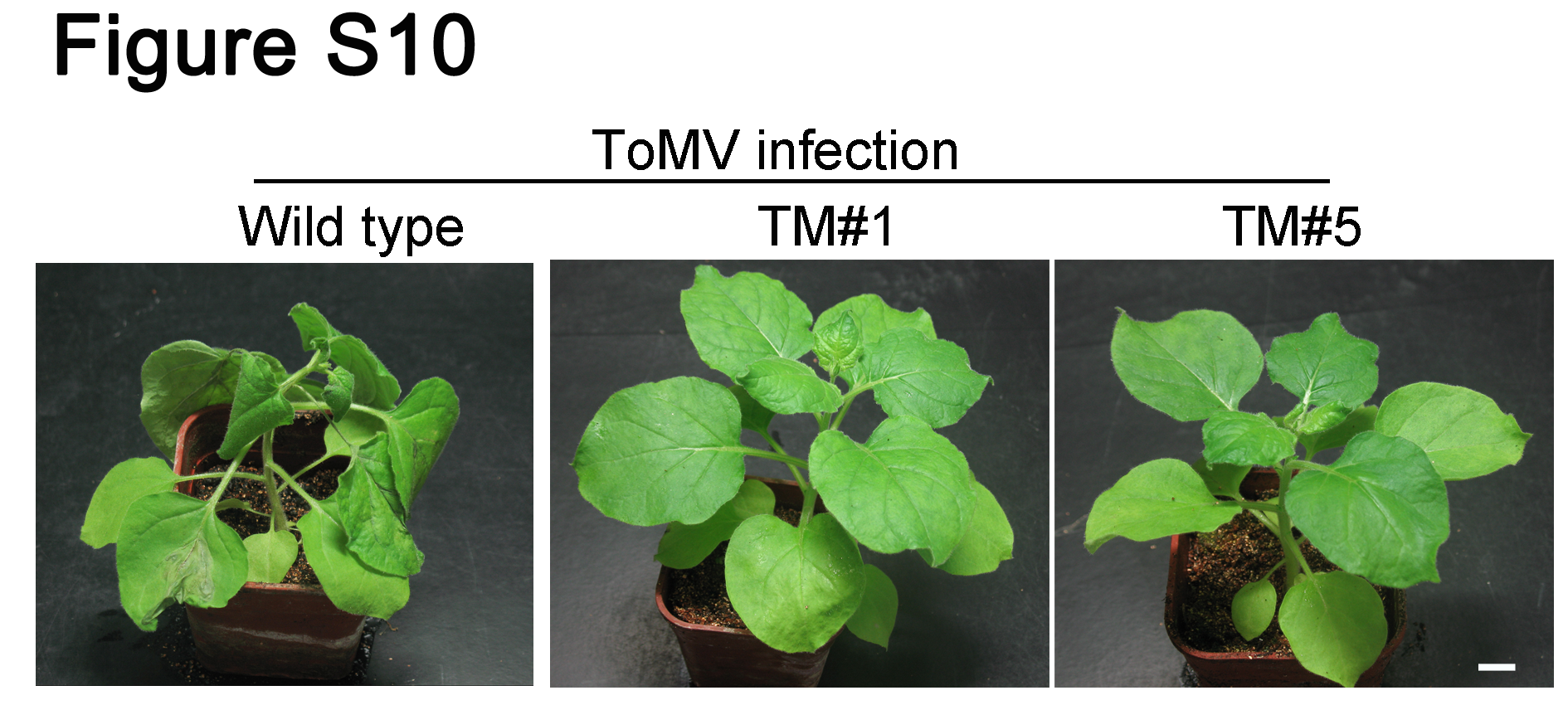

Supplement: Figure S10 — Tm-22 transgenic N. benthamiana lines TM#1 and TM#5 confer extreme resistance against ToMV. The shoots of the wild type N. benthamiana plant, but not TM#1 and TM#5 plants, became curled 7 days post ToMV infection (dpi). Scale bars represent 1 cm. (TIF) [file ppat.1003659.s010.tif]

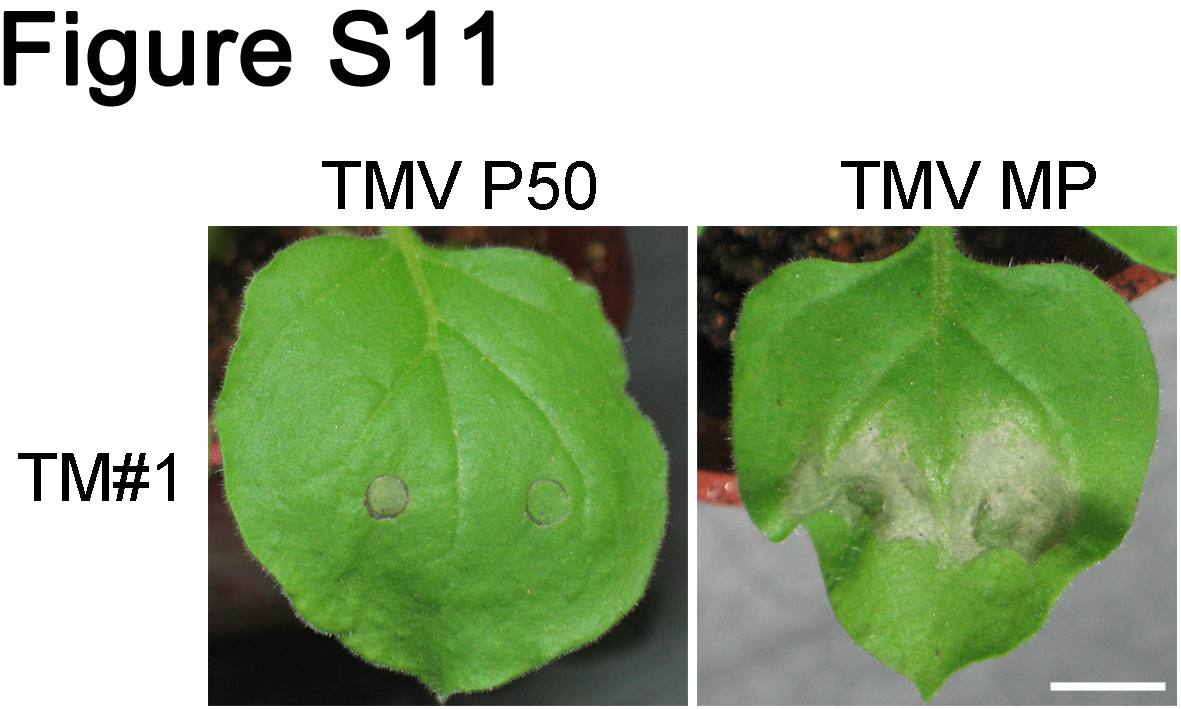

Supplement: Figure S11 — TMV MP specifically induced the HR in Tm-22 transgenic N. benthamiana line TM#1. TMV MP, but not helicase domain (P50) of TMV replicase, can induce an HR when expressed in the Tm-22 transgenic N. benthamiana line. (TIF) [file ppat.1003659.s011.tif]

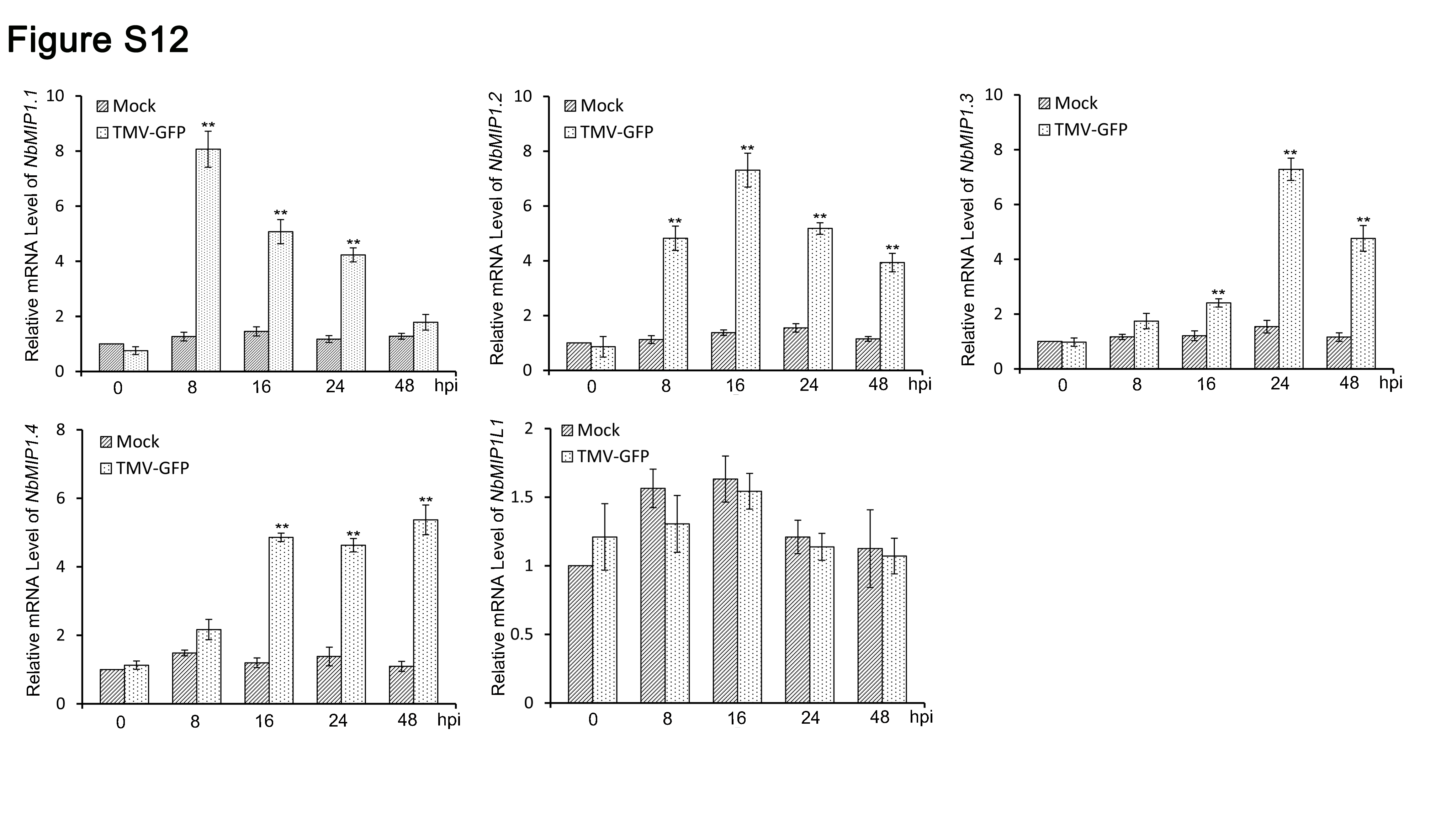

Supplement: Figure S12 — NbMIP1s were induced during Tm-22 -mediated TMV resistance in N. benthamiana . Real-time RT-PCR showing the NbMIP1s mRNA level increased after TMV-GFP infection in TM#1 plants. Actin was used as the internal control. Data are shown as means ± SD for 3 independent triplicate experiments (**P<0.01, Student's t-test). (TIF) [file ppat.1003659.s012.tif]

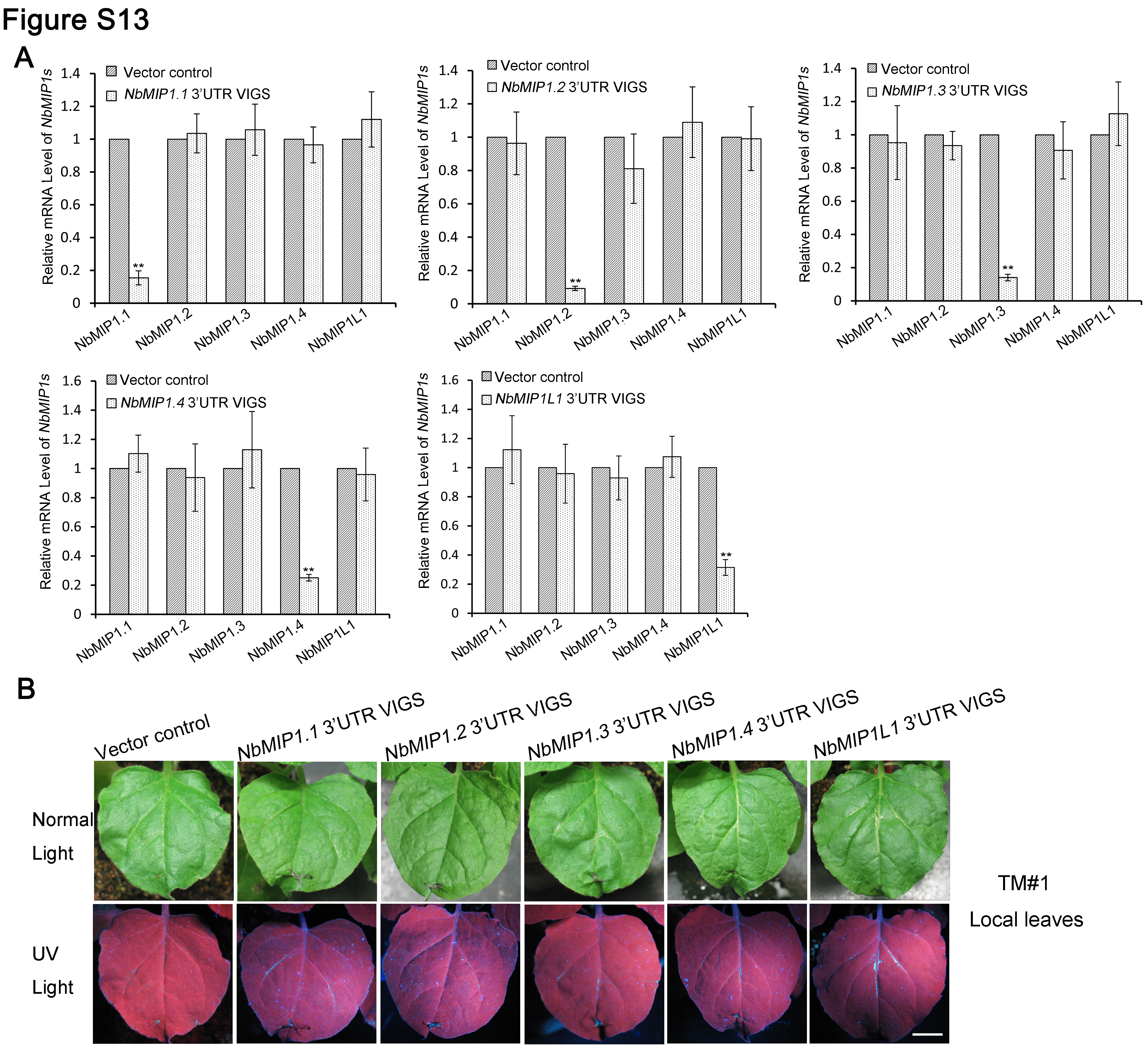

Supplement: Figure S13 — Silencing of individual NbMIP1s had no effect on Tm-22 -mediated resistance against TMV infection. (A) Real time RT-PCR to confirm the specific suppression of NbMIP1s, with Actin mRNA levels as an internal control. Data are shown as means ± SD for 3 independent triplicate experiments (**P<0.01, Student's t-test). (B) Silencing of individual NbMIP1s using their gene-specific 3′-UTRs had no effect on Tm-22-mediated resistance against TMV infection. Neither TMV-GFP infection foci nor visible HR lesions were observed in the inoculated leaves of individual NbMIP1s silenced TM#1 plants. Scale bars represent 1 cm. (TIF) [file ppat.1003659.s013.tif]

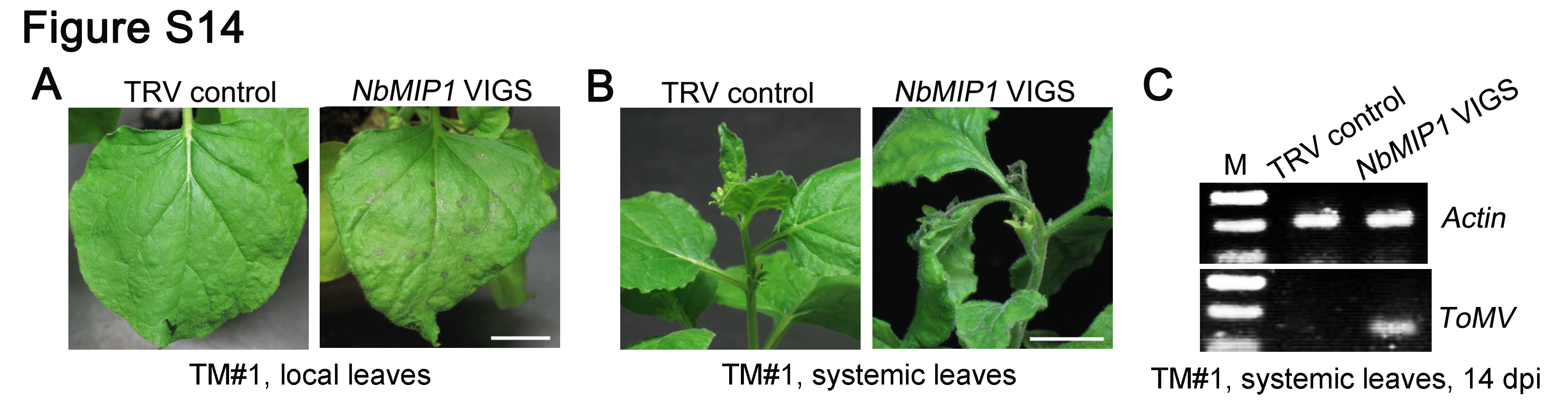

Supplement: Figure S14 — NbMIP1s are required for Tm-22 -mediated resistance against ToMV. (A) Silencing of NbMIP1s caused the appearance of visible necrotic lesions in inoculated leaves of Tm-22 transgenic N. benthamiana TM#1 plants 4 days post ToMV infection (dpi). (B) ToMV induced systemic necrosis in NbMIP1s-silenced but not in non-silenced TRV control TM#1 plants 14 dpi. Scale bars represent 1 cm. (C) RT-PCR to confirm the presence of ToMV in systemic leaves of NbMIP1s-silenced TM#1 plants. (TIF) [file ppat.1003659.s014.tif]

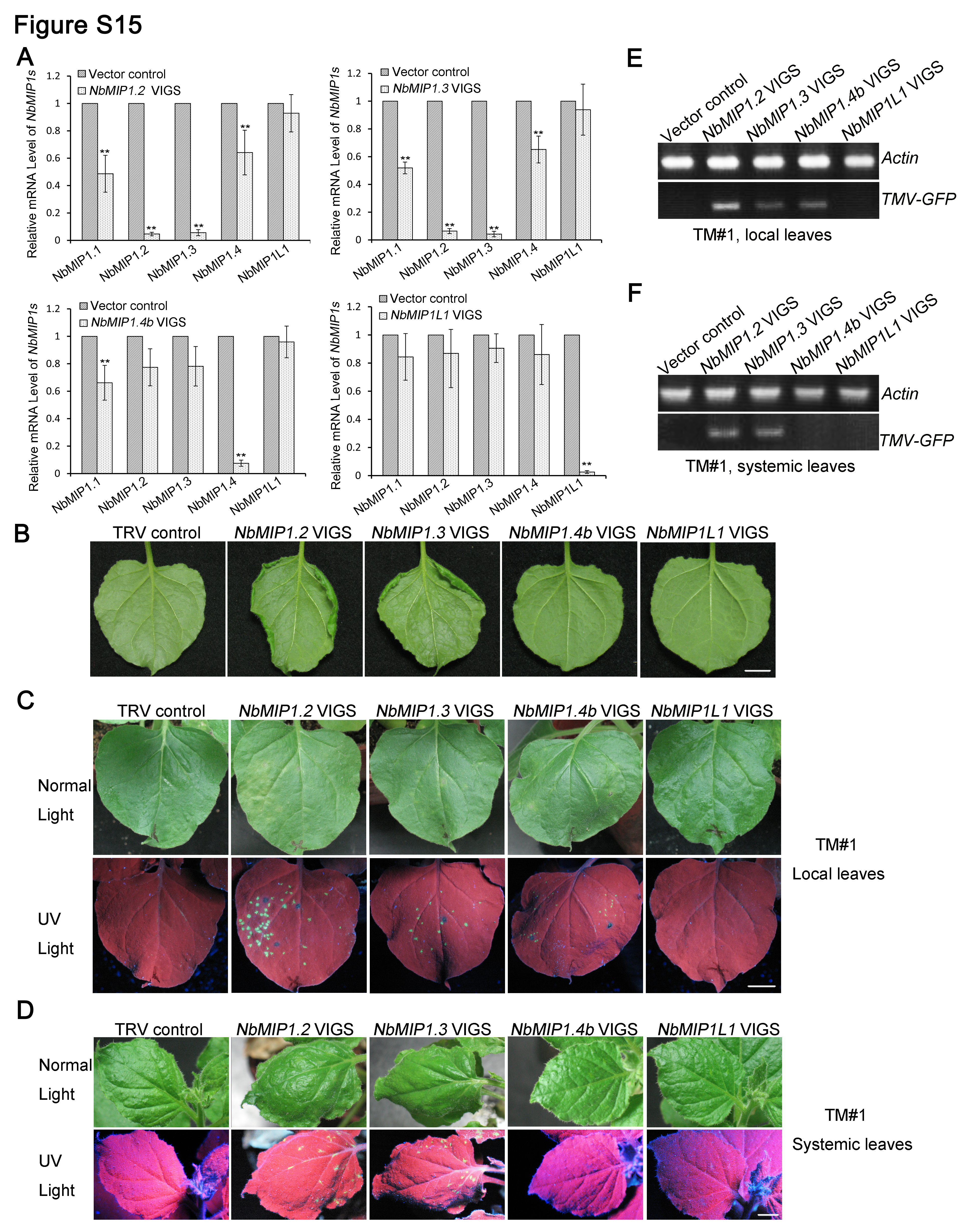

Supplement: Figure S15 — NbMIP1s are required for Tm-22 -mediated resistance against TMV-GFP. (A) Real time RT-PCR to confirm the suppression of NbMIP1s using the coding sequences of NbMIP1s; Actin mRNA levels were used as the internal control. (B) Silencing of NbMIP1s using the coding sequence of NbMIP1.2 (NbMIP1.2 VIGS) or NbMIP1.3 (NbMIP1.3 VIGS) caused downward-curled leaves. (C) Silencing of NbMIP1s using the coding sequence of NbMIP1.2, NbMIP1.3 and NbMIP1.4b (NbMIP1.4b VIGS) caused the appearance of TMV-GFP infection foci and visible HR lesions in the inoculated leaves of NbMIP1s-silenced Tm-22-containing TM#1 plants. Scale bars represent 1 cm. (D) Silencing of NbMIP1s using the coding sequence of NbMIP1.2 and NbMIP1.3 compromised Tm-22-mediated resistance against TMV, and TMV-GFP spread from the inoculated leaves into the upper non-inoculated leaves. TRV-infected TM#1 plants were used as negative controls. Photos were taken at 16 dpi. Scale bars represent 1 cm. (E–F) RT-PCR was performed to confirm the presence of TMV-GFP in local leaves (E) and systemic leaves (F) in NbMIP1.2 VIGS and NbMIP1.3 VIGS TM#1 plants. (TIF) [file ppat.1003659.s015.tif]

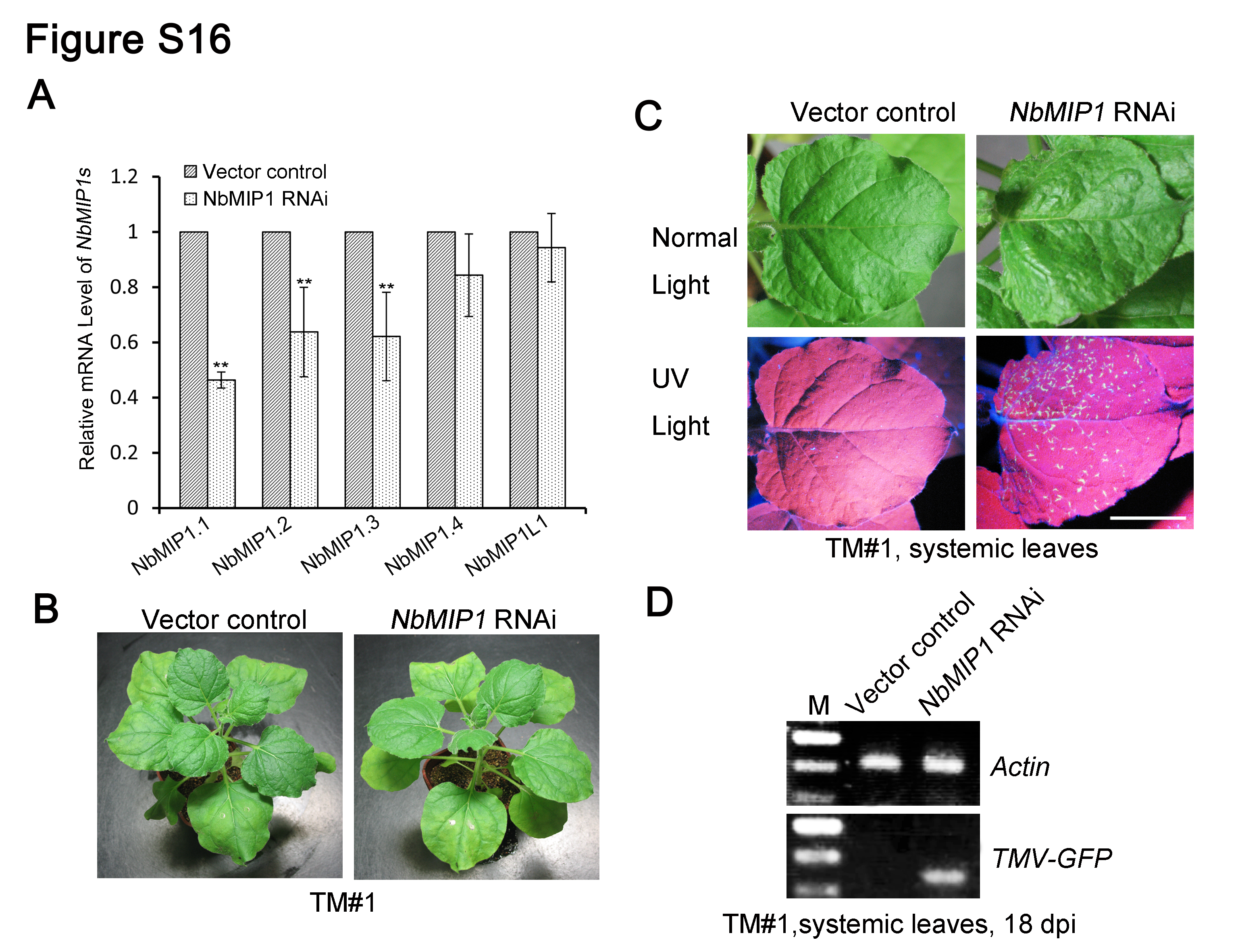

Supplement: Figure S16 — Silencing of NbMIP1s with NbMIP1 hairpin RNAi construct by agroinfiltration compromised Tm-22 -mediated resistance against TMV. (A) Real-time RT-PCR confirmed the suppression of NbMIP1s in NbMIP1 hairpin RNAi TM#1 plants, and Actin mRNA levels were used as the internal control. Data are shown as means ± SD for 3 independent triplicate experiments (**P<0.01, Student's t-test). (B) Compared to the vector control plants, NbMIP1 hairpin RNAi TM#1 plants have no visible developmental phenotype. (C) Silencing of NbMIP1s using NbMIP1 hairpin RNAi compromised Tm-22-mediated resistance against TMV, and caused TMV-GFP spreading into the upper non-inoculated leaves of TM#1 plants. The empty RNAi vector infected TM#1 plants was used as the negative control. Photos were taken at 18 dpi. Scale bars represent 1 cm. (D) RT-PCR was performed to confirm the presence of TMV-GFP in systemic leaves of NbMIP1 hairpin RNAi TM#1 plants. (TIF) [file ppat.1003659.s016.tif]

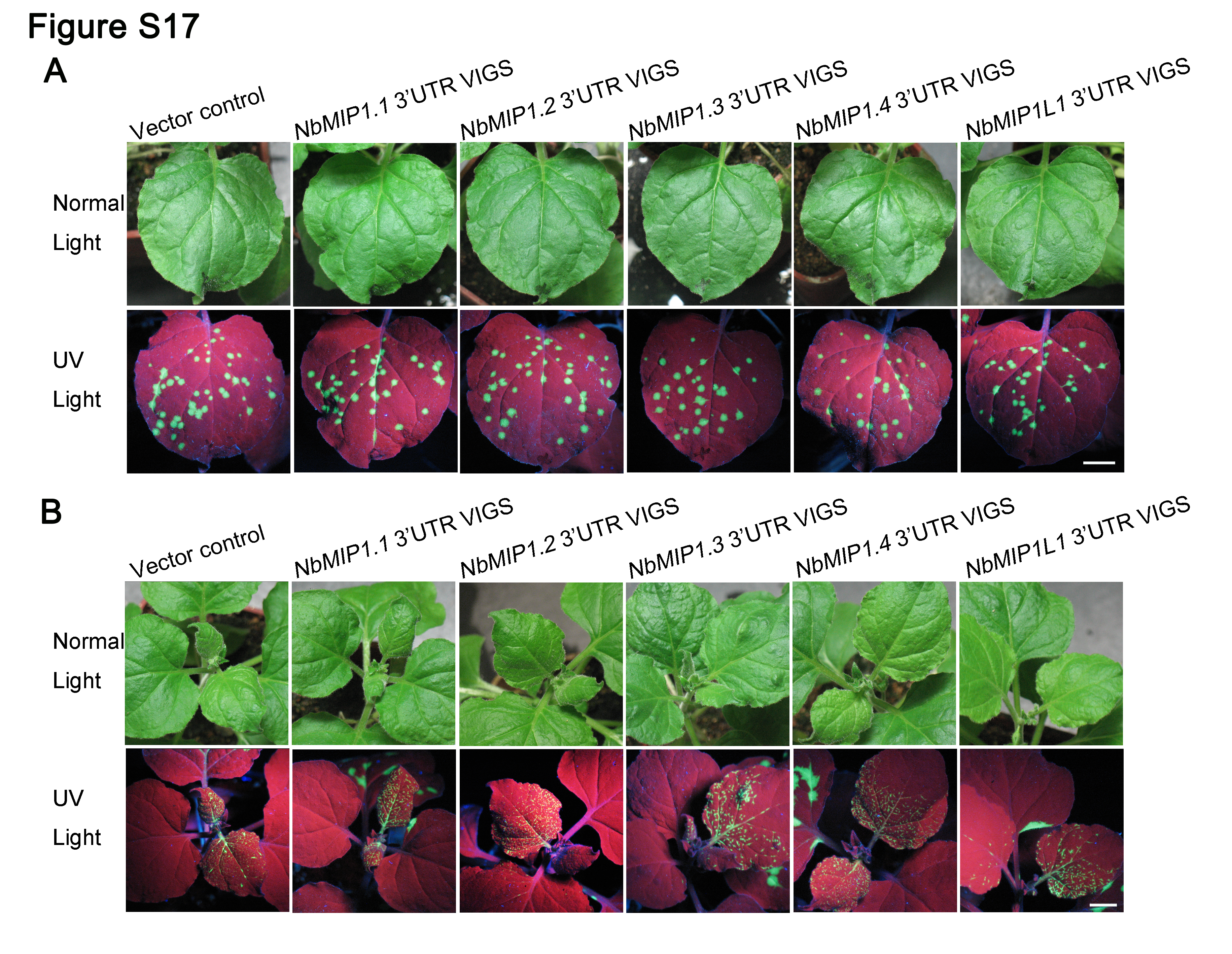

Supplement: Figure S17 — Silencing of individual NbMIP1s did not affect TMV infection. (A–B) Silencing of individual NbMIP1s using their gene-specific 3′-UTR had no effect on cell-to-cell movement (A) and systemic movement (B) of TMV. Photos were taken at 3 dpi and 5 dpi respectively. Scale bars represent 1 cm. (TIF) [file ppat.1003659.s017.tif]

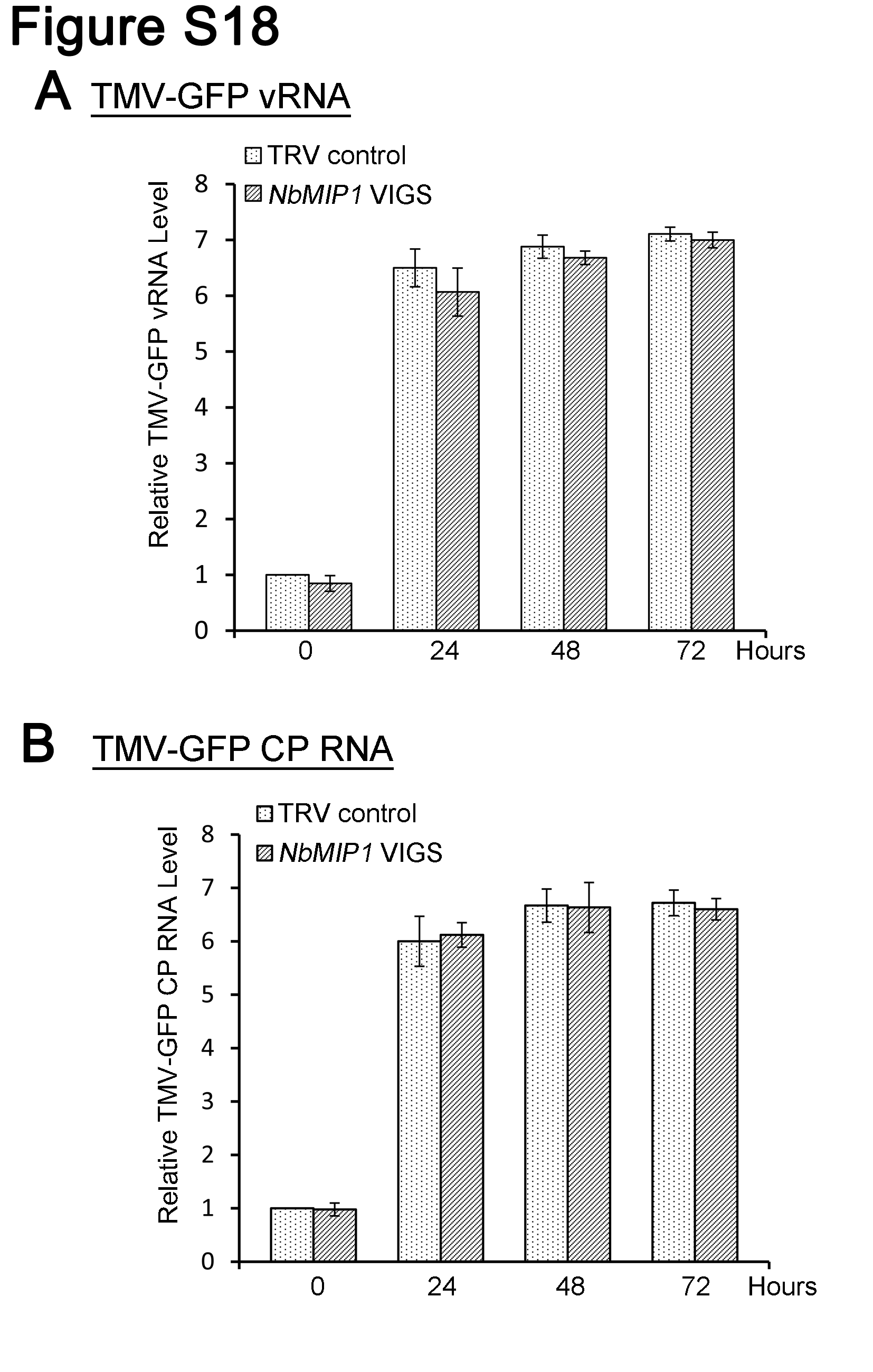

Supplement: Figure S18 — Silencing NbMIP1s had no effect on TMV replication. Real-time RT-PCR showed that TMV-GFP effectively replicated in protoplasts derived from N. benthamiana plants during TMV infection, but suppression of NbMIP1s (NbMIP1 VIGS) had no significant effect on either TMV-GFP genomic vRNA level (A) or TMV-GFP CP vRNA level (B). Actin mRNA level was used as the internal control. Data are shown as means ± SD for 3 independent triplicate experiments (Student's t-test). (TIF) [file ppat.1003659.s018.tif]

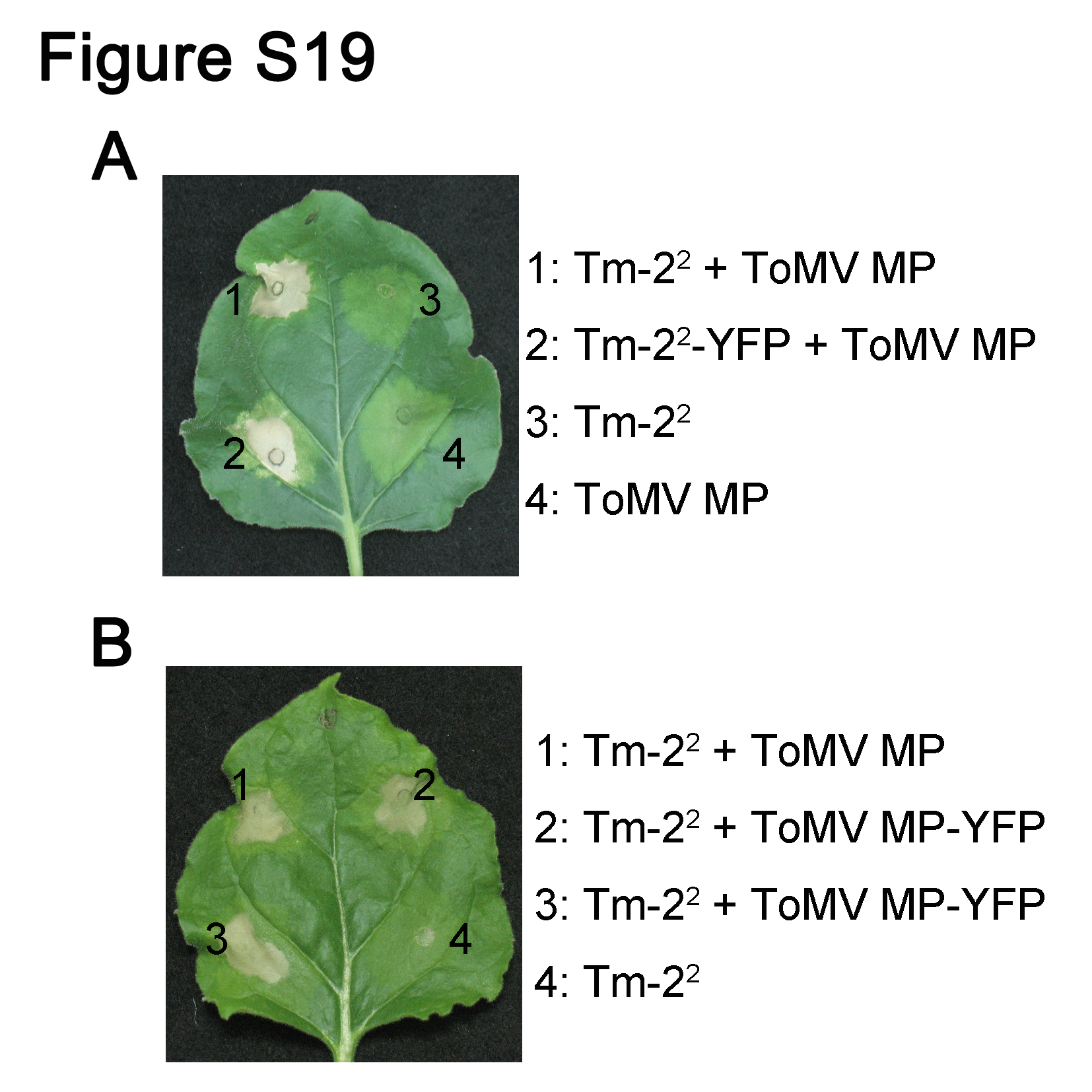

Supplement: Figure S19 — Tm-22-YFP and ToMV MP-YFP can induce the HR. (A) C-terminal YFP tagged Tm-22 (Tm-22-YFP) induced HR when co-expressed with ToMV MP. Tm-22 plus ToMV MP, Tm-22 and ToMV MP were used as positive and negative controls, respectively. (B) C-terminal YFP tagged ToMV MP (ToMV MP-YFP) induced HR when co-expressed with Tm-22. Tm-22 plus ToMV MP and Tm-22 served as positive and negative controls, respectively. (TIF) [file ppat.1003659.s019.tif]

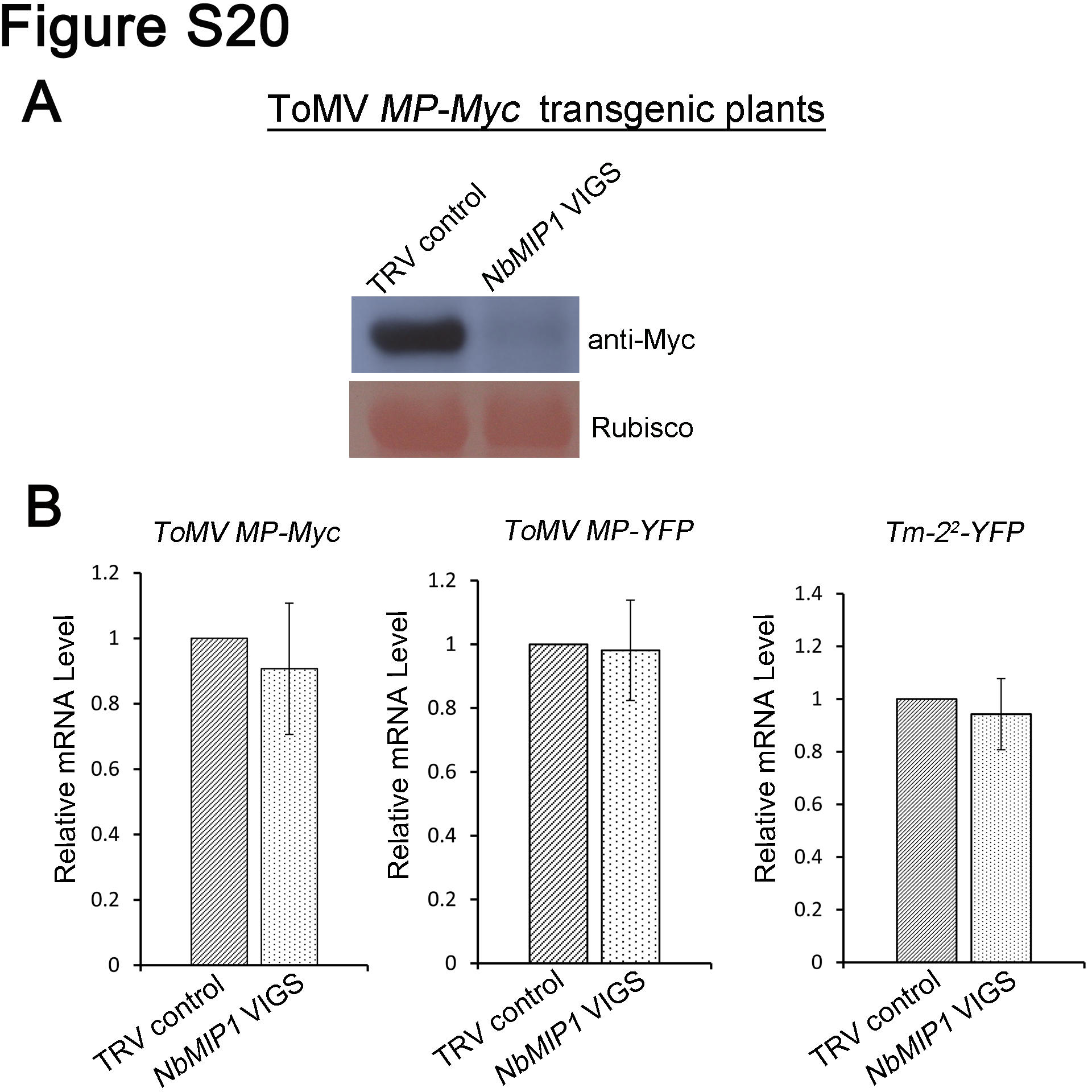

Supplement: Figure S20 — NbMIP1s are essential for the stability of ToMV MP in transgenic ToMV MP-Myc plants. (A) Silencing of NbMIP1s greatly reduced ToMV MP protein levels in transgenic plants constitutively expressing ToMV MP-Myc. Proteins were extracted from the leaf tissues of NbMIP1s-silenced and TRV-only infected transgenic ToMV MP-Myc plants respectively at 14 days post agroinfiltration for VIGS, and then followed by western blot analysis with anti-Myc antibody. Ponceau Red staining of RuBisCO indicates equal loading (lower panel). All experiments were performed three times with three replicated samples in each experiment. (B) The real-time RT-PCR showed that silencing of NbMIP1s had no effect on the mRNA level of MP-Myc, MP-YFP and Tm-22-YFP, and Actin mRNA levels were used as internal controls. Data are shown as means ± SD for 3 independent triplicate experiments (Student's t-test). (TIF) [file ppat.1003659.s020.tif]

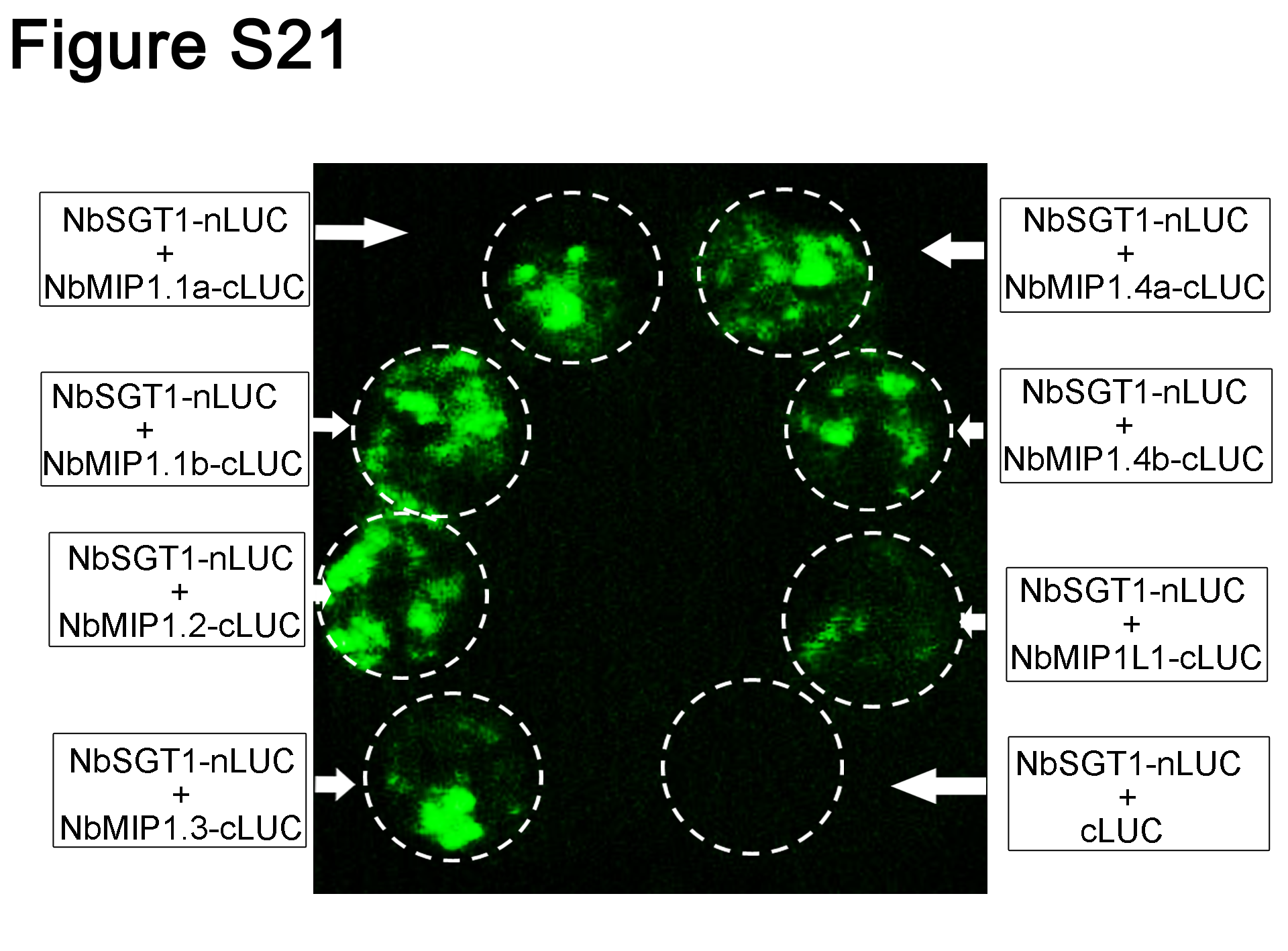

Supplement: Figure S21 — LCI assays show that NbMIP1s interact with NbSGT1 in plants. Images shown are luminescence of N. benthamiana leaves that were agro-infiltrated with NbSGT1-nLUC and NbMIP1s-cLUC. The combination of NbSGT1-nLUC and cLUC was included as the negative control. (TIF) [file ppat.1003659.s021.tif]
